# Temperature, Oxygen, and Salt-Sensing Neurons in *C. elegans* Are Carbon Dioxide Sensors that Control Avoidance Behavior

**DOI:** 10.1016/j.neuron.2011.02.023

**Published:** 2011-03-24

**Authors:** Andrew Jonathan Bretscher, Eiji Kodama-Namba, Karl Emanuel Busch, Robin Joseph Murphy, Zoltan Soltesz, Patrick Laurent, Mario de Bono

**Affiliations:** 1 MRC Laboratory of Molecular Biology, Hills Road, Cambridge CB2 0QH, UK

## Abstract

Homeostatic control of body fluid CO_2_ is essential in animals but is poorly understood. *C. elegans* relies on diffusion for gas exchange and avoids environments with elevated CO_2_. We show that *C. elegans* temperature, O_2_, and salt-sensing neurons are also CO_2_ sensors mediating CO_2_ avoidance. AFD thermosensors respond to increasing CO_2_ by a fall and then rise in Ca^2+^ and show a Ca^2+^ spike when CO_2_ decreases. BAG O_2_ sensors and ASE salt sensors are both activated by CO_2_ and remain tonically active while high CO_2_ persists. CO_2_-evoked Ca^2+^ responses in AFD and BAG neurons require cGMP-gated ion channels. Atypical soluble guanylate cyclases mediating O_2_ responses also contribute to BAG CO_2_ responses. AFD and BAG neurons together stimulate turning when CO_2_ rises and inhibit turning when CO_2_ falls. Our results show that *C. elegans* senses CO_2_ using functionally diverse sensory neurons acting homeostatically to minimize exposure to elevated CO_2_.

## Highlights

► The major temperature, O_2_, and salt-sensing neurons of *C. elegans* are CO_2_ sensors ► AFD, BAG, and ASE neurons have unique CO_2_-response properties ► O_2_-sensing atypical soluble guanylate cyclases also mediate CO_2_ neuronal responses ► CO_2_ sensing involves both transient and persistent neuronal responses

## Introduction

As the major by-product of oxidative metabolism, CO_2_ is ubiquitous in nature. Although CO_2_ comprises only ∼0.038% of Earth's atmosphere, it can accumulate to higher levels in environments with high respiration rates (Lahiri and Forster, 2003). Organisms have evolved CO_2_-sensing mechanisms to monitor both external and internal CO_2_ concentrations, but how these systems function to control physiology and behavior remain poorly understood.

Mice can smell environmental CO_2_ concentrations as low as 0.066% CO_2_ using specialized olfactory neurons that express carbonic anhydrase II (Hu et al., 2007). Carbonic anhydrases catalyze hydration of CO_2_ to generate H^+^ and HCO_3_^−^. HCO_3_^−^ is thought to stimulate the mouse olfactory neurons by activating a guanylate cyclase, GC-D (Hu et al., 2007; Sun et al., 2009). In humans the GC-D homolog is a pseudogene, and we cannot smell CO_2_ (Young et al., 2007). However, we can taste CO_2_ in carbonated solutions via sour-sensing cells on our tongues (Chandrashekar et al., 2009). In rodents, CO_2_ levels of 10% or more elicit an innate fear response in which animals freeze and avoid open spaces (Ziemann et al., 2009). This response requires activation of the acid-sensing ion channel ASIC-1A in cells of the amygdala (Ziemann et al., 2009). High concentrations of inhaled CO_2_ also modulate wakefulness by stimulating midbrain neurons (Williams et al., 2007; Richerson, 2004; Buchanan and Richerson, 2010).

Insects also sense and respond to environmental CO_2_. *Drosophila* adults and larvae avoid CO_2_ levels as low as 0.1% (Suh et al., 2004; Faucher et al., 2006). Like the CO_2_-evoked fear behavior in mice, *Drosophila* CO_2_ avoidance is innate (Suh et al., 2004) and may be part of an alarm response: stressed flies release 3- to 4-fold more CO_2_ than unstressed flies (Suh et al., 2004). *Drosophila* senses gaseous CO_2_ using two olfactory receptors, *Gr21a* and *Gr63a*, which are expressed in antennal sensory neurons (Jones et al., 2007; Kwon et al., 2007). Like other insect olfactory receptors, these do not have homologs in vertebrates or worms (Vosshall and Stocker, 2007). Artificial activation of the *Gr21a/Gr63a*-expressing neurons elicits an avoidance response (Suh et al., 2007). Whether the *Gr21a/Gr63a* receptor binds molecular CO_2_ or a CO_2_ derivative is not known. Interestingly, some food-associated odorants inhibit *Gr21a/Gr63a* CO_2_ receptor function, and the presence of food reduces CO_2_ avoidance (Turner and Ray, 2009). Although *Drosophila* avoids gaseous CO_2_, it is attracted to carbonated substrates, a response mediated by HCO_3_^−^-sensitive neurons in the proboscis (Fischler et al., 2007).

Besides monitoring external CO_2_, many animals also monitor internal CO_2_. Internal CO_2_ levels are regulated by respiratory gas exchange (Lahiri and Forster, 2003; Feldman et al., 2003; Bustami et al., 2002), but when left unregulated can lead to toxic changes in body fluid pH and death (Richerson, 2004). Mammalian respiratory CO_2_ chemoreception occurs in the brain and carotid bodies (Lahiri and Forster, 2003). The molecular mechanisms are unclear, but CO_2_-sensitive cells express carbonic anhydrases (Coates et al., 1998; Cammer and Brion, 2000), and changes in extracellular or intracellular pH modulate signaling via H^+^-sensitive ion channels (Lahiri and Forster, 2003; Richerson et al., 2005; Buckler et al., 2000; Feldman et al., 2003; Richerson, 2004; Jiang et al., 2005). Insects achieve respiratory gas exchange by opening and closing spiracles, but the control mechanisms involved are not known (Hetz and Bradley, 2005; Lehmann and Heymann, 2005).

Many small animals, including the nematode *C*. *elegans*, lack a specialized respiratory system and use diffusion for gas exchange. As in other animals, high CO_2_ levels are toxic (Sharabi et al., 2009). *C*. *elegans* appears to control internal CO_2_ by avoiding environments where this gas exceeds ∼0.5%. Avoidance requires cGMP-gated ion channels containing the TAX-2 and TAX-4 subunits (Bretscher et al., 2008; Hallem and Sternberg, 2008). Also implicated are the BAG sensory neurons, required for acute avoidance of a high CO_2_ and low O_2_ mixture (Hallem and Sternberg, 2008). Recent work indicates that the BAG neurons are transiently activated when ambient O_2_ levels fall below 10% (Zimmer et al., 2009).

Here, we show that the *C*. *elegans* head sensory neurons AFD, BAG, and ASE are primary CO_2_ sensors. AFD, BAG, and ASE were previously only known to detect changes in temperature, O_2_, and salt ion levels, respectively. Using Ca^2+^ imaging, we describe the CO_2_ responses of these neurons, which include ON, OFF, and perduring responses. We show that some, but not all, of the Ca^2+^ responses to CO_2_ depend on a cGMP-gated ion channel. Finally, we dissect how the *C*. *elegans* CO_2_ sensory system regulates CO_2_-evoked behavior. We find that the contribution of different sensors to behavior varies widely, depending on both context and stimulus dynamics.

## Results

### Multiple Sensory Neurons Mediate *C*. *elegans* Avoidance of CO_2_

**Figure 1:**
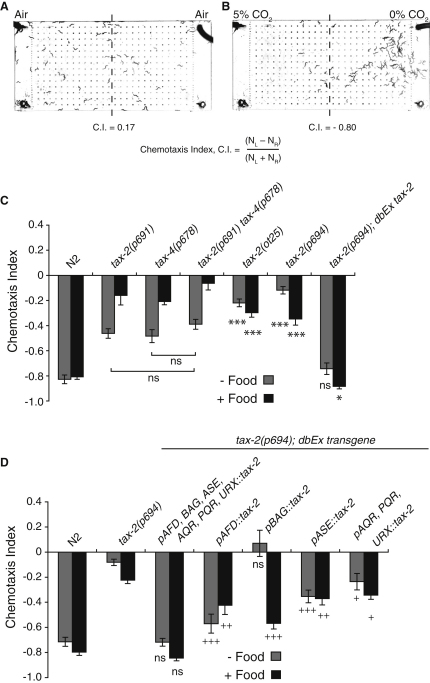
The cGMP-Gated Ion Channel Subunit TAX-2 Acts in Multiple Neurons to Promote CO_2_ Avoidance (A and B) Wild-type *C*. *elegans* distribute uniformly in air (A) but avoid 5% CO_2_ (B) over 10 min. N_L_ and N_R_, the number of animals in the left and right halves of the chamber, respectively. (C) Mutations in the cGMP-gated ion channel subunits *tax-2* and *tax-4* disrupt avoidance of 5% CO_2_ both on and off food. The null allele *tax-2(ot25)* and promoter deletion allele *tax-2(p694)* almost completely abolish CO_2_ avoidance. A transgene containing *tax-2* genomic DNA restores CO_2_ avoidance to *tax-2(p694)* mutants. In this and all subsequent figures, unless otherwise stated: a 5%-0% CO_2_ gradient was used; data points represent an average of nine assays; error bars indicate standard error of the mean (SEM); ns, not significantly different; ^∗∗∗^ or +++ indicates p < 0.001; ^∗∗^ or ++ indicates p < 0.01; ^∗^ or + indicates p < 0.05; and significance comparisons were made using the two-tailed Student's t test. (D) Expressing full-length *tax-2* cDNA in the AFD, BAG, ASE, and AQR, PQR, and URX neurons rescues CO_2_ avoidance in *tax-2(p694)* mutants. Expressing *tax-2* cDNA from the neuron-specific promoters *pgcy-8* (AFD), *pflp-17* (BAG), and an ASE-specific *pflp-6* fragment or *pgcy-32* (AQR, PQR, URX) gives varying degrees of rescue. Significance comparison of the transgenic line expressing *tax-2* in all six neurons is with N2. For all other genotypes, significance markers indicate comparison with *tax-2(p694)*.

When placed in a 5%-0% CO_2_ gradient, *C*. *elegans* migrate away from high CO_2_ (Figures 1A and 1B) (Bretscher et al., 2008). We used this assay to identify potential CO_2_-sensing neurons. Mutants defective in either the TAX-4 α or TAX-2 β cGMP-gated ion channel subunits show reduced CO_2_ avoidance, both in the presence and absence of *E*. *coli* food (Figure 1C) (Bretscher et al., 2008; Hallem and Sternberg, 2008). The defects of *tax-2*; *tax-4* double mutants recapitulated those of single mutants (Figure 1C), consistent with α and β subunits functioning together. *tax-2* and *tax-4* are coexpressed in 14 of 40 *C*. *elegans* sensory neuron classes (White et al., 1986; Komatsu et al., 1996; Coburn and Bargmann, 1996), implicating a subset of these neurons in CO_2_ sensing. A *tax-2* promoter mutation, *tax-2(p694)*, also disrupted CO_2_ avoidance (Figure 1C). Previous work reported that this allele deletes exon 1 and ∼1.6 kb of *tax-2* upstream sequences (Coburn and Bargmann, 1996). However, our sequencing data suggest that it removes only 365 bp in this interval (details in Supplemental Experimental Procedures available online). *tax-2(p694)* mutants have deficits in behaviors mediated by the AFD, BAG, ASE, AQR, PQR, and URX neurons but appear wild-type for responses mediated by other *tax-2* expressing neurons (Dusenbery et al., 1975; Hedgecock and Russell, 1975; Coburn and Bargmann, 1996; Coates and de Bono, 2002). Selectively expressing *tax-2* cDNA in AFD, BAG, ASE, AQR, PQR, and URX in *tax-2(p694)* mutants restored CO_2_ avoidance to the same extent as a full-length *tax-2* genomic fragment (Figures 1C and 1D). We next attempted to rescue the *tax-2*(*p694)* defect by expressing *tax-2* cDNA from neuron-specific promoters, confirming appropriate expression by polycistronic constructs that coexpress *tax-2* and *gfp* (Coates and de Bono, 2002). Expressing *tax-2* cDNA in the AFD thermosensory neurons strongly rescued CO_2_ avoidance, both on and off food (Figure 1D). In contrast, restoring *tax-2* to the BAG O_2_-sensing neurons rescued CO_2_ avoidance on food, as shown previously (Hallem and Sternberg, 2008), but not off food. Expressing *tax-2* cDNA in the ASE taste neurons or in the AQR, PQR, and URX O_2_-sensing neurons also partially rescued CO_2_ avoidance, both on food and off food (Figure 1D). These data implicate functionally diverse sensory neurons in CO_2_ avoidance.

### The AFD Thermosensory Neurons Sense CO_2_

**Figure 2:**
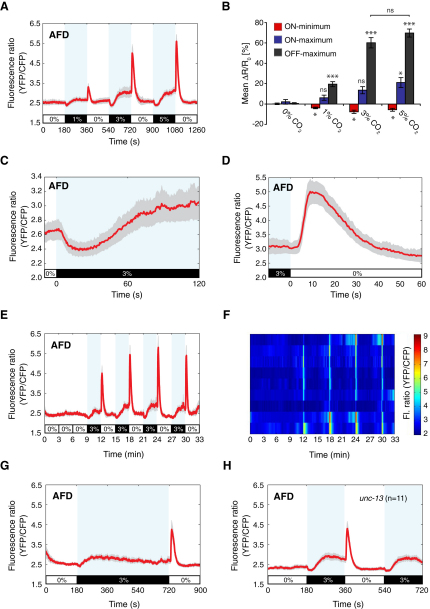
The AFD Thermosensory Neurons Sense CO_2_ (A) Mean fluorescence ratio (YFP/CFP) of AFD neurons expressing cameleon YC3.60 across a 0%-1%-0%-3%-0%-5%-0% CO_2_ stimulus. In this and all subsequent figures, blue shading indicates presence of CO_2_, and gray shading indicates the SEM (n = 26 traces). (B) Mean ratio change, *ΔR*, expressed as a percentage of the initial fluorescence ratio, *R_0_*, for the AFD ON and OFF responses for mock, 1%, 3%, and 5% CO_2_ concentrations. *ΔR = R_f_ − R_0_*, where *R_f_* is the fluorescence ratio after gas shift. Time intervals for calculation of *R_f_*, and corresponding intervals for *R_0_*, were chosen according to peaks in ratio change. For ON-minima, a 20 s time interval was used, for ON-maxima a 30 s interval, and for OFF-maxima an 8 s interval. Data for 1%, 3%, and 5% steps from (A); data for mock step from (E). Details for 1%, 3%, and 5% steps: ON-minima (150–170 s and 190–210 s, 510–530 s, and 550–570 s, 870–890 s and 910–930 s, used for *R_0_* and *R_f_*, respectively); ON-maxima (150–180 s and 330–360 s, 510–540 s, and 690–720 s, 870–900 s, and 1050–1080 s, used for *R_0_* and *R_f_*, respectively); OFF-maxima (344–352 s and 368–376 s, 704–712 s and 728–736 s, 1064–1072 s and 1088–1096 s, used for *R_0_* and *R_f_*, respectively). Significance markers indicate comparisons with responses to a mock 0% CO_2_ gas switch. Error bars indicate SEM. (C and D) Expanded view of mean AFD response to a 0%-3% CO_2_ increase (C) and 3%-0% CO_2_ decrease (D). Data from (A). (E and F) Mean AFD response to multiple 3% CO_2_ stimuli (E) and individual responses (F) plotted in a heat map (n = 9 traces). (G) Mean AFD response to a 3% CO_2_ stimulus lasting 9 min (n = 9 traces). (H) Mean AFD response to 3% CO_2_ in an *unc-13* mutant.

The AFD neurons are transiently activated when temperatures exceed cultivation levels (Kimura et al., 2004; Clark et al., 2006). To test whether AFD also responds to CO_2_, we monitored AFD intracellular Ca^2+^ levels during CO_2_ exposure using the ratiometric Ca^2+^ sensor cameleon YC3.60, expressed in AFD under control of the *gcy-8* promoter (Yu et al., 1997). Animals expressing the Ca^2+^ sensor retained wild-type CO_2_ responses (Figure S1A; see Experimental Procedures). To deliver CO_2_ stimuli, we used a Y-shaped microfluidic chamber that enables the gas phase over an immobilized animal to be switched in less than 3 s (Persson et al., 2009). In all experiments, O_2_ was maintained at 21%, with nitrogen (N_2_) completing the balance. AFD Left and AFD Right neurons responded equally to CO_2_ (Figure 2A; data not shown). On CO_2_ exposure the AFD neurons exhibited a fall in intracellular Ca^2+^ that slowly reversed to rise above baseline levels (“CO_2_-ON” response) within 2 min of CO_2_ coming on (Figures 2A and 2C). Thus, the AFD CO_2_-ON response has two components to it, an “ON-minimum” and an “ON-maximum.” Strikingly, AFD also responded to removal of CO_2_ with a fast Ca^2+^ spike that peaked within 10 s (“CO_2_-OFF” response, Figures 2A and 2D). The OFF-maximum was the largest feature of the AFD Ca^2+^ pattern, being on average 3- to 4-fold greater than the ON-maximum (Figure 2B). All three components of the AFD CO_2_ response were concentration dependent (Figure 2B). To exclude the possibility that the observed activity could be due to AFD temperature sensing, we exposed animals to 0%-0%-0% CO_2_ mock switches. Under these conditions AFD gave no responses (first 9 min, Figure 2E).

We next examined whether repeated stimulation altered AFD Ca^2+^ responses. Some *C*. *elegans* sensory neurons, such as the ALM anterior touch neurons, habituate upon repeated stimulation (Kindt et al., 2007). The AFD OFF response remained undiminished upon repeated exposure to 3% CO_2_ (Figures 2E, 2F, and S1B). We also asked whether prolonged CO_2_ exposure affects AFD responses. After a 9 min exposure to 3% CO_2_, the ON-maximum had decayed to baseline levels, whereas the OFF-maximum was unaltered (Figure 2G).

CO_2_-evoked activity in AFD could be due to synaptic input to AFD. To test this, we imaged CO_2_ responses in *unc-13* mutants, which have severe defects in synaptic release (Richmond et al., 1999). The AFD CO_2_ responses of *unc-13* animals were indistinguishable from wild-type (Figures 2H and S1C). These data suggest that, as well as being a thermosensory neuron (Mori and Ohshima, 1995; Kimura et al., 2004; Clark et al., 2007), AFD is a CO_2_ sensor with both ON and OFF responses. The sensory endings of AFD have many finger-like projections, potentially providing a large surface for CO_2_ and temperature reception (Ward et al., 1975).

AFD only responds to a temperature rise above the cultivation temperature (Kimura et al., 2004; Clark et al., 2006). If AFD temperature and CO_2_-sensing are distinct, AFD might be expected to respond to CO_2_ at temperatures below the cultivation temperature. To test this, we built a temperature-controlled stage (see Supplemental Experimental Procedures). In animals grown at 22°C, AFD responded to CO_2_ both at 15°C and at 22°C (Figures S1E and S1F). The shape of the response was similar at the two temperatures but smaller at 15°C than at 22°C. These data support the idea that AFD CO_2_ and temperature-sensing pathways are at least partly distinct.

### The BAG O_2_ Sensory Neurons Sense CO_2_

Recent work has shown that the BAG neurons are transiently activated when O_2_ levels drop below 10% (Zimmer et al., 2009). Hallem and Sternberg (2008) showed that feeding animals lacking the BAG neurons have reduced avoidance of a 10% CO_2_/10% O_2_ mixture. We have previously shown that O_2_ responses can modulate CO_2_ avoidance (Bretscher et al., 2008). These data suggest that either BAG responds exclusively to O_2_ but modulates neural circuits mediating CO_2_ responses or that BAG is a primary sensor of both O_2_ and CO_2_.

**Figure 3:**
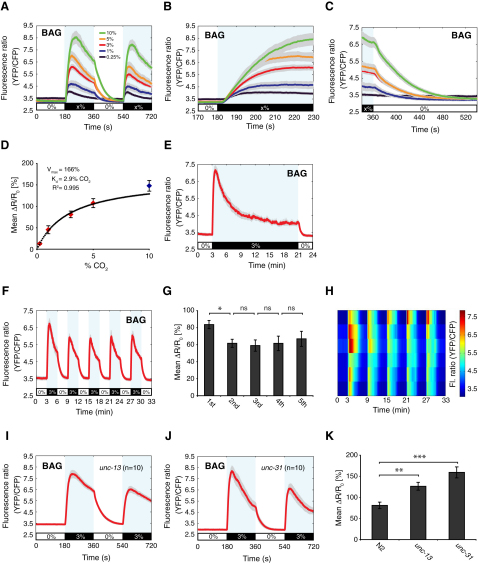
The BAG Neurons Are Highly Sensitive to CO_2_ (A–C) The BAG neurons exhibit a large “CO_2_-ON” response. Mean BAG responses to 0%-x%-0%-x% CO_2_ stimuli for x = 0.25%, 1%, 3%, 5%, or 10% CO_2_. Shown are the full response (A), a 60 s interval across CO_2_ introduction (B), and a 180 s interval across CO_2_ removal (C) (n = 10 or more traces for all concentrations). (D) Dose-response curve for the BAG CO_2_ response. The mean fluorescence ratio change, *ΔR*, is plotted as a percentage of the mean baseline fluorescence ratio, *R_0_*. *ΔR = R_f_ − R_0_*. *R_f_* was calculated from the peak of the BAG Ca^2+^ response at 200–260 s and *R_0_* from 120–180 s. Curve fit of the standard equation for a single-site binding process (Michaelis-Menten, y *=* V_max_x/(K_d_ + x), where V_max_ and K_d_ are constants with units of [% mean ratio change] and % CO_2_, respectively) to the red data points using least-squares regression analysis. The blue data point (10% CO_2_) was omitted from the curve fit because at 10% CO_2_ the BAG fluorescence ratio (YFP/CFP) falls outside of the linear dynamic range of the Ca^2+^ sensor YC3.60. Curve fit gives K_d_ = 2.9% CO_2_, and V_max_ = 166% mean *ΔR/R_0_*, with a goodness of fit R^2^ regression value of 0.995. Error bars indicate SEM. (E) Mean BAG response to a 3% CO_2_ stimulus lasting 18 min (n = 9 traces). (F–H) BAG responses to a 0%-3%-0%-3%-0%-3%-0%-3%-0%-3%-0% CO_2_ stimulus. (F) Mean fluorescence ratio (YFP/CFP), (G) mean percent (%) *ΔR/R_0_*, and individual BAG Ca^2+^ traces plotted in a heat map (H) (n = 6 traces). (I–K) Mean BAG Ca^2+^ responses in *unc-13* mutants (I) and *unc-31* mutants (J). (K) Mean percent (%) *ΔR/R_0_* values for (I) and (J). Asterisks indicate significance compared to wild-type.

To test BAG neuron CO_2_ sensitivity, we created animals expressing cameleon YC3.60 in BAG from a *pflp-17::YC3.60* transgene and imaged Ca^2+^ levels. The BAGL and BAGR neurons were exquisitely sensitive to a rise in CO_2_ (Figures 3A–3C). Cameleon reported a rise in Ca^2+^ that peaked after ∼30 s and then decayed (Figures 3A and 3B). The excitability threshold of BAG was below 0.25% CO_2_. A plot of mean fluorescence ratio change against percent (%) CO_2_ suggests that BAG reaches half-maximal activity at ∼2.9% CO_2_ (Figure 3D). Thus, BAG neurons respond to both O_2_ and CO_2_.

Elevated CO_2_ persistently stimulates locomotory activity in feeding *C*. *elegans*, suggesting that some CO_2_-sensing circuits can signal tonically in high CO_2_ (Bretscher et al., 2008). During prolonged high CO_2_ the BAG Ca^2+^ spike decayed to a plateau that persisted until CO_2_ removal, at which point Ca^2+^ returned to resting levels (Figure 3E). Thus, BAG exhibits both a transient peak and a perduring Ca^2+^ plateau in response to elevated CO_2_. As with AFD, we asked whether BAG neurons habituate. During five stimulus cycles of 3% CO_2_, BAG showed a decrement in response amplitude after the first CO_2_ stimulus, but no habituation thereafter (Figures 3F–3H).

To test if the BAG neurons are primary CO_2_ sensors, we disrupted synaptic input to BAG using the *unc-13* and *unc-31* mutations. *unc-31* mutants are defective in dense-core vesicle release, but not synaptic vesicle release (Speese et al., 2007). Neither the *unc-13* nor the *unc-31* mutations disrupted BAG Ca^2+^ responses, suggesting that BAG neurons are intrinsically CO_2_ sensitive (Figures 3I–3K). However, the magnitude of Ca^2+^ responses in these mutants was significantly enhanced, particularly in *unc-31* animals, suggesting that BAG activity is normally inhibited by neuromodulators.

### The Asymmetric ASEL and ASER Taste Neurons Are Both Activated by CO_2_

We next examined CO_2_ responses in the ASE neurons that mediate chemotaxis to water-soluble cues, including salt ions such as Na^+^ and Cl^−^ (Bargmann and Horvitz, 1991; Ortiz et al., 2009). ASEL and ASER are functionally asymmetric (Hobert et al., 2002). ASEL is activated by a rise in the concentration of NaCl, whereas ASER is activated by a drop (Suzuki et al., 2008). For NaCl responses, activation of ASEL inhibits animals from reversing, whereas activation of ASER increases reversal likelihood (Suzuki et al., 2008).

**Figure 4:**
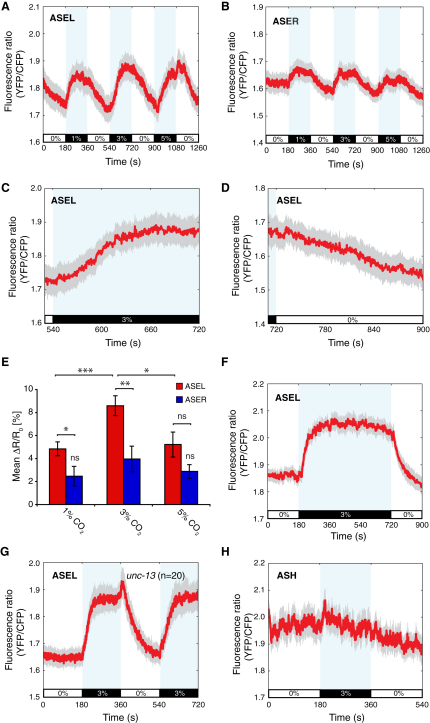
The ASEL and ASER Neurons Are Activated by CO_2_ (A and B) Mean responses of ASEL (A) and ASER (B) to 0%-1%-0%-3%-0%-5%-0% CO_2_. ASEL, n = 26 traces; ASER, n = 19 traces. (C and D) Expanded view of mean ASEL response to a 0%-3% CO_2_ increase (C) and 3%-0% CO_2_ decrease (D). (E) Mean percent (%) *ΔR*/*R_0_* for ASEL and ASER for 1%, 3%, and 5% CO_2_ stimuli. Baseline ratio, *R_0_*, was calculated from the 60 s before CO_2_ exposure, and peak ratio, *R_f_*, was calculated from the 60 s before CO_2_ removal. ASER responses to 1% and 5% CO_2_ are not significantly different from responses to 3% CO_2_. ASEL responses to 1% and 5% CO_2_ are significantly different from responses to 3% CO_2_. ASEL responses are significantly different from ASER responses for 1% and 3% CO_2_, but not 5% CO_2_. (F) Mean ASEL response to a 3% CO_2_ stimulus lasting 9 min (n = 14 traces). (G) Mean ASEL response to 3% CO_2_ in an *unc-13* mutant. (H) The ASH neurons are not activated by 3% CO_2_ (n = 21 traces).

We imaged ASEL and ASER Ca^2+^ responses to CO_2_, using animals expressing the Ca^2+^ sensor YC2.12 in ASE from a *pflp-6::YC2.12* transgene (Suzuki et al., 2008). Both ASEL and ASER were activated by 1%, 3%, and 5% CO_2_ (Figures 4A–4E), although the responses of ASEL were generally ∼2-fold larger than those of ASER (Figure 4E). ASE responses to CO_2_ were slow, taking around 2 min for Ca^2+^ levels to peak (Figure 4F). Sustained elevated CO_2_ led to sustained increases in Ca^2+^ (Figure 4F). As for AFD and BAG, ASE neurons appeared to be intrinsically CO_2_ sensitive because Ca^2+^ responses were intact in *unc-13* mutants (Figures 4G and S1D). In summary, ASEL and ASER both respond to CO_2_ by a slow rise in Ca^2+^ that persists while CO_2_ is high and returns to baseline when CO_2_ returns to baseline.

### AQR, PQR, and URX O_2_-Sensing Neurons Are Weakly CO_2_ Responsive

We examined whether the AQR, PQR, and URX O_2_-sensing neurons (Persson et al., 2009; Zimmer et al., 2009) respond to CO_2_ because our *tax-2* rescue data indicated that these neurons contribute, albeit weakly, to CO_2_ avoidance. Average Ca^2+^ traces indicated that unlike AFD, BAG, and ASE, none of these neurons respond reliably to CO_2_ (Figures S2A–S2D). URX most consistently showed CO_2_-evoked activity, and this was retained in *unc-13* mutants (Figures S2A, S2E, and S2F). AQR and PQR occasionally showed a Ca^2+^ rise associated with an increase in CO_2_ but also showed apparent spontaneous activity that lay out of synchrony with the CO_2_ stimulus (Figures S2B–S2D). The response of PQR to a 0%-3%-0%-3% CO_2_ stimulus was dwarfed by its response to a 21%-11%-21%-11% O_2_ stimulus (Figure S2C).

Having identified three *C*. *elegans* neuron classes that responded strongly to CO_2_ and a further three that responded weakly to CO_2_, we considered the possibility that all sensory neurons show some CO_2_ responsiveness. Therefore, we imaged Ca^2+^ responses to CO_2_ in the ASH neurons that respond to various aversive stimuli (Hilliard et al., 2005). ASH showed no response to 3% CO_2_ (Figure 4H). This suggests that AFD, BAG, and ASE are functionally specialized as CO_2_ sensors.

### CO_2_ Sensitivity in BAG and AFD Requires a cGMP-Gated Ion Channel

**Figure 5:**
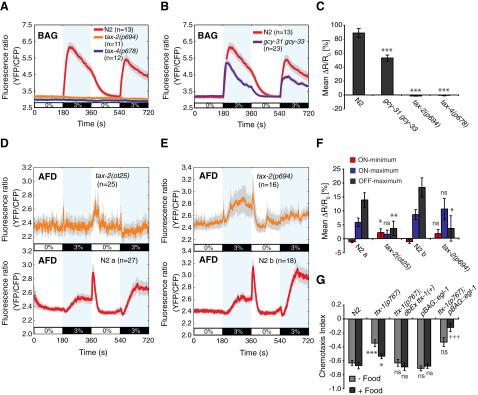
A cGMP Pathway Couples CO_2_ to BAG and AFD Activation, and These Neurons Are Required for CO_2_ Avoidance (A) Mutations in the *tax-2* and *tax-4* cGMP-gated ion channel subunits abolish BAG responses to 3% CO_2_. (B) Mean BAG responses to 3% CO_2_ in wild-type and *gcy-31(ok296) gcy-33(ok232)* double-mutant animals. (C) Mean percent (%) *ΔR/R_0_* values for the BAG responses in (A) and (B). (D and E) Mean AFD response to 3% CO_2_ of *tax-2(ot25)* null (D) and *tax-2(p694)* promoter deletion (E) mutants and their wild-type controls. Longer exposure times were used in imaging AFD in *tax-2(ot25)* animals due to weak expression of YC3.60. (F) Mean percent (%) *ΔR/R_0_* values for the AFD ON-minima, ON-maxima, and OFF-maxima of *tax-2(ot25)*, *tax-2(p694)*, and wild-type. Significance markers indicate comparisons against wild-type. (G) AFD and BAG both contribute to CO_2_ avoidance in shallow spatial gradients. *ttx-1* mutants have defects in CO_2_ avoidance both on and off food. These defects are fully rescued by *ttx-1(+)* genomic DNA. Genetic ablation of BAG alone does not disrupt CO_2_ avoidance, but loss of BAG when AFD is absent further disrupts CO_2_ avoidance on food. Asterisks (^∗^) and “ns” indicate significance comparisons against N2 wild-type. Plus signs (+) and “ns” indicate significance comparisons against *ttx-1(p767)* mutants.

Our *tax-2* rescue data suggested that CO_2_ sensing in BAG and AFD neurons involves cGMP signaling. To examine this further we imaged BAG responses to CO_2_ in *tax-2(p694)* and *tax-4(null)* mutants. Both mutations completely abolished CO_2_-evoked Ca^2+^ responses in BAG (Figures 5A and 5C). This suggests that BAG CO_2_ sensory transduction is mediated by TAX-2/TAX-4 cGMP-gated channels and by extension, upstream guanylate cyclases (*gcy*).

The only *gcy* genes known to be expressed in BAG are the atypical soluble guanylate cyclases *gcy-31* and *gcy-33* (Yu et al., 1997; Zimmer et al., 2009; Ortiz et al., 2006). These appear to be O_2_ regulated (Gray et al., 2004; Boon and Marletta, 2005) because both are required for BAG O_2_ responses (Zimmer et al., 2009). To examine if GCY-31, GCY-33, or both are required in CO_2_ sensory transduction, we imaged BAG responses to 3% CO_2_ in *gcy-31; gcy-33* double-deletion mutants. Loss of *gcy-31* and *gcy-33* reduced the CO_2_-evoked BAG Ca^2+^ response (Figures 5B and 5C). This suggests that GCY-31 and/or GCY-33 forms part of the CO_2_ sensory system in BAG, although other molecules are likely to be involved.

We next imaged AFD responses in *tax-2(null)* and *tax-2(p694)* animals. Expression from the *gcy-8* promoter is markedly reduced in *tax-2* and *tax-4* mutants (Satterlee et al., 2004), and YC3.60 expression was correspondingly low in AFD in *tax-2(ot25null)* animals. In contrast, expression in *tax-2(p694)* animals was similar to wild-type (data not shown). Both *tax-2* mutations significantly reduced the AFD CO_2_ response, but neither completely abolished it (Figures 5D–5F). The AFD ON-minimum appeared to be absent in both *tax-2* mutants, whereas the AFD ON-maximum was absent in *tax-2(null)* animals but enhanced in *tax-2(p694)* animals (Figures 5D–5F). Our data suggest that all three components of the AFD CO_2_ response involve TAX-2 mediated cGMP pathways but that other pathways also contribute.

### *C*. *elegans* Carbonic Anhydrases Are Expressed in Several Neurons, Including BAG

To further investigate molecular mechanisms of CO_2_ sensing, we asked whether *C*. *elegans* CO_2_ sensors express carbonic anhydrases, hallmarks of CO_2_-responsive neurons in other animals (Hu et al., 2007; Wang et al., 2002; Ridderstrale and Hanson, 1985; Coates et al., 1998). Database searches indicate that the *C*. *elegans* genome encodes eight predicted carbonic anhydrases. Six, *cah-1* to *cah-6*, belong to the alpha family, and two, *bca-1* and *bca-2,* to the beta family. Because many members of the beta family are mitochondrial (Syrjänen et al., 2010; Fasseas et al., 2010), we focused our studies on the alpha family. We fused upstream promoter regions of each gene to *gfp* and examined the resulting expression patterns. We found that *cah-1*, *2*, *3*, and *6* show strong neuronal expression in adults (Figure S3A). *cah-4* was primarily expressed in the hypodermis (excluding the seam cells) and in the excretory cell, consistent with a kidney-like function for this cell. *cah-3* and *cah-5* show expression in intestinal cells, with *cah-3* expression being especially strong. Using a *pBAG::mCherry* marker, we showed that *cah-2*, but not apparently any of the other five *cah* genes, was expressed in BAG (Figure S3B). *cah-2* was also expressed in a set of four quadrant head neurons, other unidentified head neurons, the canal neurons CANL/R, whose processes run parallel to the tracts of the excretory cell, and a pair of tail neurons (Figure S5). Previous data suggest that *cah-2* is also expressed in AFD (Colosimo et al., 2004). These data suggest that BAG and AFD neurons are specialized CO_2_ sensors that coexpress carbonic anhydrases and CO_2_-regulated cGMP pathways. They also raise the possibility that other *C*. *elegans* neurons and tissues respond to CO_2_.

### AFD and BAG Direct Avoidance Behavior in Spatial CO_2_ Gradients

To investigate how CO_2_ sensors contribute to avoidance in spatial gradients, we genetically ablated neurons. We focused on AFD and BAG neurons because the Ca^2+^ responses of ASE to CO_2_ stimuli were slow, and those of AQR, PQR, and URX, weak. Specification of the AFD neurons requires the *otd*/*Otx* homeodomain transcription factor *ttx-1*, which is expressed only in AFD (Satterlee et al., 2001). *ttx-1* mutants show thermotactic defects equivalent to those of animals in which AFD has been removed by laser ablation (Mori and Ohshima, 1995). *ttx-1* mutants had a strong CO_2_ avoidance defect off food, and a weaker defect on food (Figure 5G). Wild-type avoidance was restored to *ttx-1* mutants by a transgene containing *ttx-1* genomic DNA (Figure 5G). These data suggest that the AFD neurons promote CO_2_ avoidance in spatial CO_2_ gradients.

To ablate BAG we expressed the *egl-1* programmed cell death activator from a BAG-specific *gcy-33* promoter (Conradt and Horvitz, 1998; Yu et al., 1997) (we thank M. Beverly and P. Sengupta for this line). Both BAGL and BAGR neurons were absent in greater than 90% of animals bearing this transgene (Table S1 available online). Surprisingly, the CO_2_ avoidance of BAG-ablated animals was not significantly different from wild-type, both on and off food (Figure 5G). We asked if combined genetic ablation of AFD and BAG causes a synthetic CO_2_ avoidance phenotype. Ablating the BAG neurons disrupted the residual CO_2_ avoidance of *ttx-1(p767)* mutants on food (Figure 5G). However, in the absence of food, *ttx-1(p767); pgcy-33::egl-1* animals showed no greater defect than *ttx-1(p767)* single mutants (Figure 5G). These data show that AFD and BAG promote CO_2_ avoidance in spatial gradients on food, and that AFD and at least one other neuron that is not BAG promote avoidance when food is absent. Thus, the importance of different sensory neurons for CO_2_ avoidance in spatial gradients depends on context.

### AFD and BAG Control Discrete Aspects of the *C*. *elegans* Response to CO_2_

**Figure 6:**
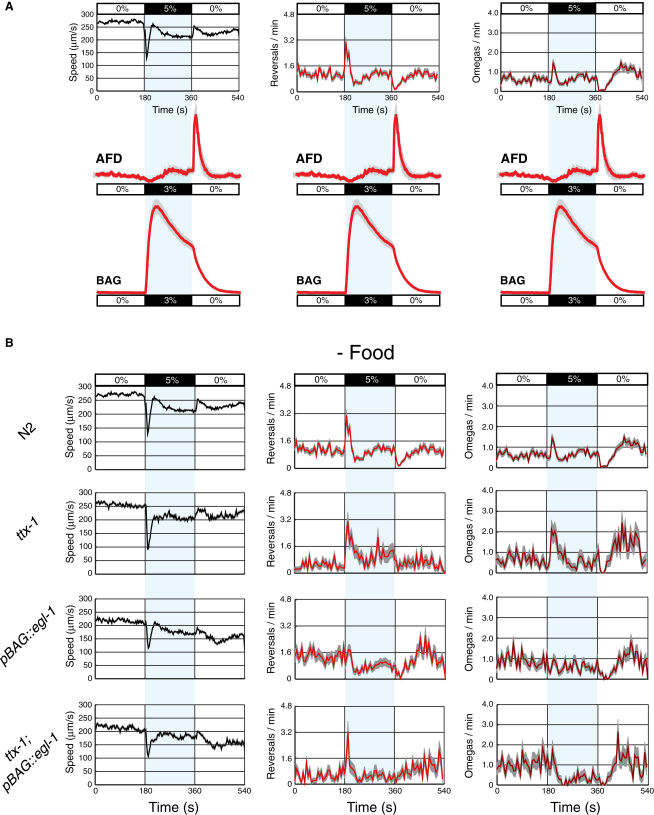
AFD and BAG Control Behavioral Responses to Changes in Percent (%) CO_2_ (A) AFD and BAG CO_2_-evoked neuronal events correlate with CO_2_-evoked behavioral events. Behavioral plots reproduced from (B). (B) Average speed, reversal, and omega rates of wild-type (N2), AFD-ablated (*ttx-1*), BAG-ablated (*pgcy-33::egl-1*), and AFD-ablated BAG-ablated (*ttx-1; pgcy-33::egl-1*) animals off food across a 0%-5%-0% CO_2_ stimulus. Stimulus bar and light blue shading indicate the timing of gas switches. Gray shading indicates SEM. Speed (μm/s, black line) calculated in 3 s bins. Reversal (orange line) and omega rates (maroon line) are in event initiations per animal per minute calculated in 6 s bins. N2, n = 59 movies; *ttx-1(p767)*, n = 20 movies; *pgcy-33::egl-1*, n = 15 movies; *ttx-1; pgcy-33::egl-1*, n = 16 movies.

In 5%-0% CO_2_ spatial gradients (Figure 1), a *C*. *elegans* moving at ∼0.3 mm/s experiences a change of 0%-0.05% CO_2_/s, depending on bearing relative to the gradient. In our Ca^2+^-imaging experiments, immobilized animals experienced much sharper temporal gradients of ∼1% CO_2_/s. In the wild, animals are likely to encounter a variety of CO_2_ gradients. To analyze behavioral responses to sharp CO_2_ gradients, we designed a square-shaped microfluidic chamber that enables CO_2_ levels over freely moving animals to be switched rapidly (Movie S1 available online). We recorded responses and used custom software to extract instantaneous speed, reversal rate, and rate of omega turns, turns in which an animal's head and tail touch to form an “Ω” shape (N2, Figure 6B). In the absence of food, a rise in CO_2_ from 0% to 5% elicited a brief slowing followed by a transient increase in reversals and omega turns (Figure 6B). A rapid drop in CO_2_, from 5% to 0%, elicited an acceleration that coincided with suppression of reversals and omega turns.

**Figure 7:**
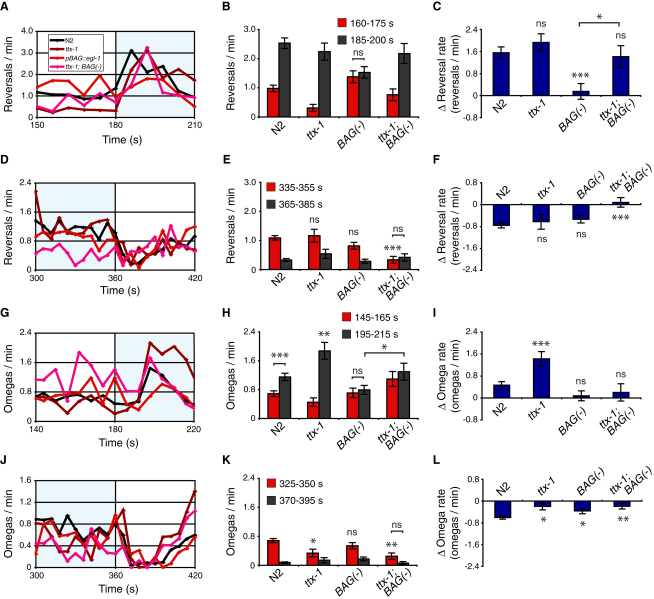
AFD and BAG Together Promote Turning When CO_2_ Levels Rise and Inhibit Turning When CO_2_ Levels Fall (A–L) Statistical analysis of reversal and omega turns of wild-type and ablated animals during 0%-5% CO_2_ increases and 5%-0% CO_2_ decreases. Average behavioral traces are shown at left, time-averaged behavioral rates before and after gas switches are shown at middle, and average changes in behavioral rates are shown at right. Rates are in initiations of reversals or omega events per animal per minute. (A, D, G, and J) Average reversal and omega rates during 0%-5% CO_2_ and 5%-0% CO_2_ gas switches. Error bars omitted for clarity. (B, E, H, and K) Time-averaged reversal (B and E) and omega (H and K) rates before (red bars) and after (dark gray bars) an increase (B and H) or a decrease (E and K) in percent (%) CO_2_. Intervals for comparison coincide with stationary points in wild-type behavioral rates. Error bars indicate SEM. (C, F, I, and L) Average change in reversal (C and F) and omega (I and L) rates across an increase (C and I) or a decrease (F and L) in percent (%) CO_2_. Difference calculations based on data in (B), (E), (H), and (K), and error bars calculated from SEM values in (B), (E), (H), and (K) using error propagation formulae. Significance markers indicate comparisons against wild-type, unless otherwise indicated.

The timing of CO_2_-evoked Ca^2+^ responses in both AFD and BAG correlated with peaks in locomotory activity (Figure 6A). We investigated these correlations directly by ablating AFD and/or BAG and examining behavioral responses (Figure 6B). For statistical comparison, we chose time intervals before and after gas switches according to the occurrence of peaks in wild-type behavioral rates. In the absence of food, neither AFD nor BAG ablation abolished modulation of speed across shifts in CO_2_ (Figures 6B and S4). Stronger phenotypes were observed for reversal and omega rates (Figure 6B). Unexpectedly, ablation of AFD increased reversal and omega rates following a sharp CO_2_ rise (*ttx-1*, Figures 6B, 7B, 7C, 7H, and 7I) and reduced suppression of omega turns following a CO_2_ fall (*ttx-1*, Figures 6B, 7K, and 7L), suggesting that AFD acts to suppress reversals and omega turns at these two time points. Ablation of BAG abolished reversal and omega responses to a rise in CO_2_ (*pBAG::egl-1*, Figures 6B, 7B, 7C, 7H, and 7I) and reduced the suppression of omega turns following a CO_2_ fall (*pBAG::egl-1*, Figures 6B, 7K, and 7L), consistent with BAG excitation promoting reversals and omega turns. Coablation of AFD and BAG abolished the suppression of reversals and omega turns following a fall in CO_2_ (*ttx-1*; *pBAG::egl-1*, Figures 7F and 7L). This effect was due to reduced reversal and omega rates under prolonged high CO_2_ (*ttx-1*; *pBAG::egl-1*, red bars, Figures 7E and 7K). These data suggest that together BAG and AFD act to suppress reversals and omega turns when CO_2_ decreases.

Curiously, AFD-ablated BAG-ablated animals continued to show a transient increase in reversals following a CO_2_ rise (*ttx-1*; *pBAG::egl-1*, Figures 6B, 7B, and 7C). This result suggests that there is at least one other CO_2_ “ON” sensory neuron, XYZ, that promotes reversals in response to a CO_2_ rise. It also suggests that after a CO_2_ rise, AFD acts antagonistically to both BAG and the hypothetical XYZ neuron to inhibit reversals. We investigated whether the ASE or AQR, PQR, URX neurons could be XYZ by ablating them together with AFD and BAG. Ablating ASEL/R had no significant effect on the reversal rate of AFD-ablated BAG-ablated animals immediately following a CO_2_ rise (*che-1; ttx-1*; *pBAG::egl-1*, Figures S5A–S5D) but did alter reversal rates under prolonged high CO_2_ (Figures S5E and S5F). The ablation of AQR, PQR, URX by an integrated *pgcy-36::egl-1* transgene caused an increase in the reversal rate of AFD-ablated BAG-ablated animals in air alone (Figures S5A–S5D). These data suggest that the ASE neurons suppress reversals under prolonged high CO_2_ and that the AQR, PQR, URX neurons suppress reversals in the absence of CO_2._ However, even animals defective in AFD, BAG, ASE, AQR, PQR, and URX retained some CO_2_ responsiveness, suggesting that *C*. *elegans* has additional CO_2_ sensors.

### The Presence of Food Modulates the Neural Circuit Controlling CO_2_ Avoidance

Wild-type *C*. *elegans* (N2) exhibit distinct locomotory patterns in the presence and absence of food (de Bono and Bargmann 1998; Sawin et al., 2000). Animals move slowly and reverse frequently on food, whereas in its absence they move rapidly with fewer reversals. The escape mechanisms elicited by a CO_2_ rise on and off food were correspondingly different (Movies S1 and S2 and Figure S6). Feeding animals still briefly slowed down when CO_2_ levels rose but then switched to a high locomotory rate as high CO_2_ persisted (Figure S6) (Bretscher et al., 2008). Coupled to the slowing response was a much stronger transient increase in omega turns (Figure S6). Feeding animals also persistently suppressed reversals in high CO_2_. These mechanisms increased the exploratory behavior of feeding animals, presumably helping them to escape from high CO_2_.

To investigate whether AFD and BAG contribute to differences between on- and off-food behavior, we ablated them. AFD ablation abolished the increased speed response to high CO_2_ and resulted in inappropriately high-reversal and omega rates under high CO_2_ (*ttx-1*, Figure S6). In contrast, ablating only BAG had little or no effect (*pBAG::egl-1*, Figure S6). Ablating neither AFD nor BAG alone abolished the dramatic spike in omega turns following a CO_2_ rise, but ablating both neurons together nearly did (*ttx-1; pBAG::egl-1*, Figure S6). As for off food, loss of AFD and BAG did not eliminate CO_2_ responses, suggesting that other neurons contribute to rapid CO_2_-evoked behavior on food.

In summary, genetic ablation suggests that AFD and BAG account for much of the different behavioral strategies employed in CO_2_ avoidance on and off food. In both contexts one or more other neurons also contribute to CO_2_ avoidance.

## Discussion

### The AFD, BAG, and ASE Sensory Neurons Exhibit Distinct CO_2_ Responses

*C*. *elegans,* like mammals, monitors CO_2_ using multiple neuron types. CO_2_ sensors include the ASE neurons with sensory endings directly exposed to the external environment and AFD and BAG neurons whose dendrites lie within the animal. All three neuron types are primary CO_2_ sensors: their CO_2_ responses are unimpaired in *unc-13* mutants defective in synaptic release. Each neuron type has a unique CO_2_ response. In AFD, a rise in CO_2_ triggers an initial drop in intracellular Ca^2+^ levels (AFD ON-minimum), then a rise above baseline (AFD ON-maximum), and when CO_2_ is removed, a spike (AFD OFF-maximum). This complexity may reflect multiple CO_2_-transduction mechanisms. In contrast, BAG and ASE neurons are activated by a rise, but not a fall, in CO_2_. In BAG, Ca^2+^ peaks within 60 s of a rise in CO_2_, then decays to a plateau that persists as long as CO_2_ remains high; Ca^2+^ drops back to baseline upon CO_2_ removal. ASE responds slowly to CO_2_ exposure: Ca^2+^ takes 2 min to peak but remains elevated while CO_2_ is high. The tonic activity of BAG and ASE neurons in high CO_2_ may allow *C*. *elegans* to modify responses to other cues, perhaps by affecting sensory pathways or interneuron networks.

AFD, BAG, and ASE also sense other stimuli. AFD senses temperature (Kimura et al., 2004), BAG senses ambient O_2_ (Zimmer et al., 2009), and ASE senses salt (Suzuki et al., 2008). This may enable sensory integration within sensory neurons. For each of the three neurons, CO_2_ and non-CO_2_ stimuli evoke distinct Ca^2+^ responses. When temperature rises above the cultivation level, AFD responds with a monophasic Ca^2+^ spike that lasts a few seconds (Kimura et al., 2004; Clark et al., 2007). The dissimilar CO_2_ and temperature responses suggest that the two stimuli are sensed differently. Supporting this, AFD responds to CO_2_ below the cultivation temperature. The Ca^2+^ responses of BAG to high CO_2_ and low O_2_ are more similar in shape (Figure 3) (Zimmer et al., 2009). In contrast, the responses of ASE to CO_2_ and NaCl differ markedly (Figure 4) (Suzuki et al., 2008). First, unlike CO_2_, NaCl evokes an asymmetric response in ASEL and ASER: a rise in NaCl triggers a Ca^2+^ spike in ASEL but a drop in Ca^2+^ in ASER. Second, ASEL/R Ca^2+^ responses to NaCl adapt rapidly, whereas sustained CO_2_ stimulation leads to sustained high Ca^2+^ in ASE (Figure 4F). Third, whereas ASE responses to CO_2_ are slow, taking around 2 min for Ca^2+^ to peak, responses to NaCl peak within 30 s of stimulus exposure. The slowness of ASE CO_2_ responses could reflect rate-limiting hydration of environmental CO_2_.

### cGMP Signaling Mediates CO_2_ Responses

CO_2_ sensing in AFD, BAG, and ASE involves cGMP signaling. Mutating the cGMP-gated channel subunit *tax-2* partially abolishes the AFD Ca^2+^ response to CO_2_ and completely abolishes CO_2_-evoked activity in BAG (Figure 5). CO_2_-evoked Ca^2+^ responses in ASE likely also depend on cGMP-gated channels because expression of *tax-2* cDNA in ASE in *tax-2* mutants partially restores CO_2_ avoidance (Figure 1). In mouse olfactory epithelia, CO_2_ sensing requires the transmembrane guanylate cyclase GC-D, which is activated by HCO_3_^−^ (Hu et al., 2007; Sun et al., 2009). The hallmarks that make GC-D HCO_3_^−^ regulated are unknown, but the *C*. *elegans* genome encodes 27 transmembrane *g*uanylate *cy*clase (*gcy*), a subset of which could be similarly regulated (Yu et al., 1997; Ortiz et al., 2006). The AFD neurons express *gcy-8*, *gcy-18*, *gcy-23*, and *gcy-29*. *gcy-8 gcy-18 gcy-23* triple mutants have a thermotaxis defect similar to that of the AFD specification mutant *ttx-1* (Inada et al., 2006), but have no defect in CO_2_ avoidance in a 5%-0% CO_2_ gradient (data not shown). ASE neurons express 11 transmembrane guanylate cyclases, nine of which are expressed asymmetrically either in ASEL or ASER (Ortiz et al., 2006).

**Figure 8:**
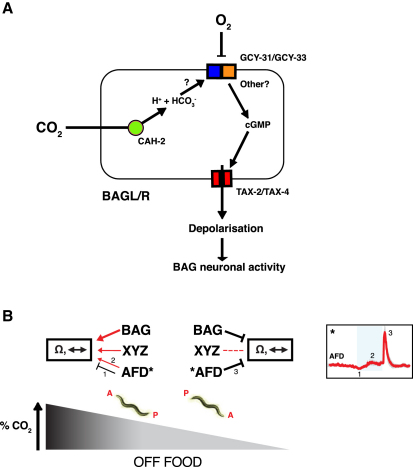
Models of CO_2_ Sensory Neuron Function (A) Model of CO_2_ and O_2_-evoked excitability in the BAG neurons. As in mouse CO_2_ olfactory neurons, a carbonic anhydrase CAH-2 catalyzes hydration of CO_2_ in BAG. HCO_3_^−^ ions or H^+^ protons may activate the GCY-31/GCY-33 heterodimer as well as another guanylate cyclase. Elevated cGMP levels open the TAX-2/TAX-4 channel causing Ca^2+^ influx and BAG depolarization. (B) AFD, BAG, and the postulated CO_2_-ON sensory neuron XYZ ensure turning probability increases when the CO_2_ gradient is positive and decreases when negative. As an animal moves up a CO_2_ gradient, increasing CO_2_ activates BAG and XYZ, which activate turning. AFD initially inhibits turning (ON-minimum, 1), but as Ca^2+^ levels rise (ON-maximum, 2), AFD promotes turning. As an animal moves down a CO_2_ gradient, a decrease in CO_2_ causes deactivation of BAG and the AFD OFF-response (3), which suppress turning. Activation is represented by red arrows, and inhibition, by black “T” symbols. The worm at left is heading up the gradient, and the worm at right, down the gradient. A, anterior; P, posterior. “Ω” represents omega turns and the double-headed arrow, reversals.

Transmembrane guanylate cyclase expression has not been reported in BAG. However, BAG expresses the atypical soluble guanylate cyclases GCY-31 and GCY-33 (Yu et al., 1997). Simultaneously disrupting *gcy-31* and *gcy-33* reduced the CO_2_-evoked Ca^2+^ response amplitudes in BAG, suggesting that GCY-31 and/or GCY-33 contribute to CO_2_ sensing. GCY-31 and GCY-33 are thought to function as heterodimers that have an O_2_-binding heme cofactor (Boon and Marletta, 2005) and are required for BAG O_2_-evoked Ca^2+^ responses when O_2_ drops below 10% (Zimmer et al., 2009). An intriguing possibility is that the GCY-31/GCY-33 heterodimer is inhibited by O_2_ and activated by CO_2_, making it a sensory integrator of CO_2_ and O_2_ signals in BAG (Figure 8A); however, we cannot rule out the possibility of a linked mutation disrupting BAG responses.

AFD, BAG, and ASE are unlikely to be the only CO_2_-responsive neurons in *C*. *elegans*. The AQR, PQR, and URX O_2_-sensing neurons showed sporadic responses to CO_2_ (Figure S2), and selective expression of *tax-2* cDNA in these neurons partially restored CO_2_ avoidance to *tax-2(p694)* mutants, suggesting that they are CO_2_ sensitive. Moreover, more than ten *C*. *elegans* neurons express carbonic anhydrases, some of which may be unidentified CO_2_ sensors.

### The Contribution of Different Sensors to CO_2_ Avoidance Varies with Stimulus Dynamics and Context

Why does *C*. *elegans* have multiple CO_2_ sensors? One reason is that sensors are deployed differently according to the dynamics of the CO_2_ stimulus. For example, when food is absent, BAG mediates responses to sharp CO_2_ gradients but is less important for navigating shallow gradients (compare Figures 5G and 6B). A second reason is that context modifies the behavioral changes needed to escape CO_2_. For example, when food is present, *C*. *elegans* move slowly and reverse frequently. To efficiently escape high CO_2_ in a food-containing environment, *C*. *elegans* increase speed and suppress reversals relative to the “on food” ground state. By contrast when food is absent, animals are already moving quickly and reversing less frequently. Correspondingly, the importance of BAG for CO_2_ avoidance depends on both stimulus shape and food context. Whereas BAG-ablated animals respond poorly to rapid CO_2_ changes when food is absent, they respond like wild-type animals when food is present (*pBAG::egl-1*, Figures 6 and S6). Conversely, in shallow gradients BAG acts redundantly with AFD to promote CO_2_ avoidance when food is present but is not important when food is absent, even when AFD is ablated (Figure 5G).

How do the Ca^2+^ responses of CO_2_ sensory neurons encode behavior? CO_2_-evoked neuronal events in AFD and BAG correlate with peaks and troughs in locomotory rates (Figure 6A). To investigate these relationships, we ablated CO_2_ sensors. One caveat of neuronal ablation is that it can only remove a neuron in its entirety, and not individual components of its responses. Ablation of AFD and BAG neurons one at a time and together suggests that: (1) BAG activation and the AFD ON-minimum act antagonistically, promoting and suppressing reversal and omega rates, respectively (Figures 7C and 7I); (2) BAG plateau activity and the AFD ON-maximum both act to promote reversal and omega rates during maintained high CO_2_ (*ttx-1; BAG(-)*, Figures 7E and 7K); and (3) decay of BAG activity and the AFD OFF-maximum act together to suppress reversals and omega turns following CO_2_ removal (*ttx-1; BAG(-)*, Figures 7F and 7L). Together our data suggest that when an animal is migrating up a CO_2_ gradient, BAG and AFD trigger turning, whereas when an animal is migrating down a CO_2_ gradient, AFD and BAG suppress turning (Figure 8B). Therefore, it appears that the three different components of the AFD CO_2_ response may differentially regulate behavior (1, 2, 3, AFD, Figure 8B). Because AFD(−) BAG(−) animals still respond to CO_2_, we also infer the existence of an additional sensory neuron, XYZ, that is neither ASE nor AQR, PQR, URX, that promotes turning when CO_2_ rises (Figure 8B).

### CO_2_ Avoidance Behavior in *C*. *elegans* Appears to Be a Homeostatic Mechanism

Elevated tissue CO_2_ is toxic (Richerson, 2004). In *C*. *elegans*, CO_2_ levels exceeding 9% disrupt body muscle organization and general development and reduce fertility (Sharabi et al., 2009). The CO_2_ responses of AFD, BAG, and ASE neurons do not habituate upon multiple exposures to CO_2_ (Figures 2 and 3; data not shown). *C*. *elegans* CO_2_ avoidance in spatial gradients is also nonhabituating over a similar period (data not shown). By contrast, *C*. *elegans* attraction to benzaldehyde (L'Etoile et al., 2002), response to noxious Cu^2+^ ion stimuli (Hilliard et al., 2005), and response to nose touch (Kindt et al., 2007) all habituate. Moreover, BAG and ASE neurons show tonic signaling while CO_2_ levels are high, at least over 20 min. We speculate that *C*. *elegans* CO_2_ avoidance habituates slowly and performs a homeostatic function by preventing CO_2_ poisoning of body tissues. *C*. *elegans* CO_2_ avoidance provides an opportunity for detailed examination of a CO_2_ homeostatic system with comparative ease relative to the systems of more complex animals.

## Experimental Procedures

### Strains

Strains were grown at 22°C under standard conditions (Brenner, 1974). Mutant combinations were made by following visible phenotypes or using PCR to confirm genotype. A full list of strains can be found in Supplemental Experimental Procedures.

### Behavioral Assays

Spatial CO_2_ gradient assays were as described (Bretscher et al., 2008). Briefly, polydimethylsiloxane (PDMS) chambers connected to gas syringe pumps were placed over adult worms on a 9 cm agar plate. After 10 min the distribution of worms was used to calculate a chemotaxis index (Figure 1). Chemotaxis bar graphs represent the average of nine independent assays performed over 3 days.

For temporal gradient assays a square 11 × 11 × 0.2 mm PDMS chamber was placed over adult worms on 6 cm agar plates. For off-food assays, ∼40 animals were picked after washing in M9 Buffer to remove adhering *E*. *coli*. For on-food assays, a 2-day-old 20 μl *E*. *coli* lawn was used. Worms were allowed to crawl on food for 1 hr. After placing the chamber, animals were left for 4 min before exposure to a 0%-5%-0% CO_2_ stimulus. Behavior was captured using a Grasshopper CCD camera (Point Grey Research). A TTL-output from a frame counter (custom built) controlled opening and closing of Teflon™ pinch valves (Automate Scientific) at defined time points, controlling the switching of gases. Worms were tracked using DIAS Software (Solltech), and worm object paths were created. The centroid X and Y coordinates, maximum length, mean width, perimeter, and roundness were extracted for each worm object across frames. From these parameters, speed, omega initiation rate, and reversal initiation rate were calculated using a custom-written program in MATLAB (The MathWorks). Omega turns were detected by circular object topologies. This method gave 90.9% success using the stringent criterion that worm head touches worm tail. Reversal events were defined as forward movement (F), followed by backward movement (B), followed by return to forward movement (F). Using the criterion of an F-B-F event and optimized parameters minimum allowable reversal angle (150°), maximum reversal duration (7.5 s), and minimum reversal distance (0.3 mm, life size), reversal detection success rate ran at 81.25%. Detection parameters were optimized by minimizing the sum of the squared differences between detection outputs of computer and a human observer for Movie S1. Behavior occurring during merger of worm objects was discarded. Temporal gradient assay data represent the average of 16 or more movies for off food and nine or more for on food.

In all experiments, percent (%) CO_2_ was balanced by percent (%) N_2_ while 21% O_2_ was maintained. In rescue experiments, transgenic animals were preselected by following coinjection markers. In all figures, statistical significance was determined using the two-tailed Student's t test.

### Calcium Imaging

Ca^2+^ imaging was on an inverted microscope (Axiovert; Zeiss), using a 40× C-Apochromat lens and MetaMorph acquisition software (Molecular Devices). Agarose pads were made in M9 Buffer (pH 6.8) and 1 mM CaCl_2_, mimicking an NGM substrate. Worms expressing the Ca^2+^ sensor YC3.60 showed wild-type avoidance in 5%-0% CO_2_ gradients (Figure S1). Worms were glued to pads using Nexaband glue (WPI Inc.) and placed under the stem of the Y-chamber microfluidic device. Photobleaching was minimized using a 2.0 optical density filter and a shutter to limit exposure time to 100 ms per frame. An excitation filter (Chroma) restricted illumination to the cyan channel. A beam splitter (Optical Insights) was used to separate the cyan and yellow emission light. The ratio of the background-subtracted fluorescence in the YFP and CFP channels was calculated with Jmalyze (Kerr and Schafer, 2006). Fluorescence ratio (YFP/CFP) plots were made in MATLAB. Movies were captured at 2 fps. Average Ca^2+^ traces were compiled from at least six recordings made on 2 or more days.

## Supplemental Information

Document S1. Six Figures, One Table, and Supplemental Experimental Procedures

Movie S1. *C. elegans* Responses to a 0%–5%–0% CO_2_ Stimulus following a 1–3–1 min Timeline off Food Animals placed within the square chamber microfluidic device are exposed to 5% CO_2_ stimuli off food. Movie speeded up 5×. Timing of CO_2_ stimulus is indicated.

Movie S2. Responses of Feeding *C. elegans* to a 0%–5%–0% CO_2_ Stimulus following a 1–3–1 min Timeline As in Movie S1, but animals are exposed to 5% CO_2_ while on a lawn of *E. coli* food. Movie speeded up 5×. Timing of CO_2_ stimulus is indicated.
