# Supplementary material for: Temperature, Oxygen, and Salt-Sensing Neurons in C. elegans Are Carbon Dioxide Sensors that Control Avoidance Behavior
Source: Neuron. 2011 Mar 24;69(6-4):1099–113. doi: 10.1016/j.neuron.2011.02.023 (PMC3115024; doi:10.1016/j.neuron.2011.02.023)
Supplement: Document S1. Six Figures, One Table, and Supplemental Experimental Procedures [file mmc1.pdf]

Neuron, volume *69*

Supplemental Information

Temperature, Oxygen, and Salt-Sensing Neurons in *C. elegans* Are Carbon Dioxide Sensors that Control Avoidance Behavior

Andrew Jonathan Bretscher, Eiji Kodama-Namba, Karl Emanuel Busch, Robin Joseph Murphy, Zoltan Soltesz, Patrick Laurent and Mario de Bono

Supplementary Figures and Figure Legends

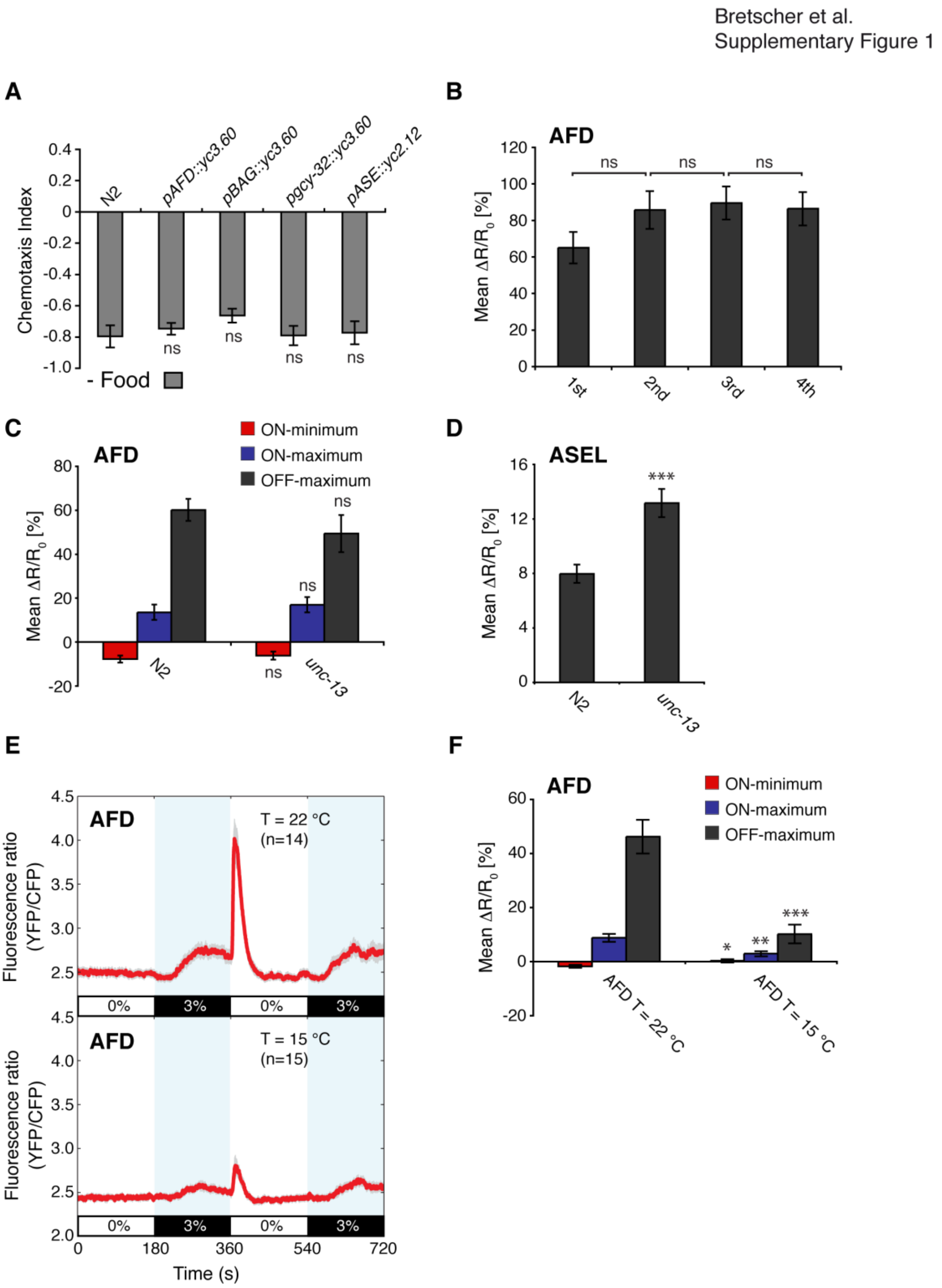

Supplementary Figure 1. (A) Cameleon YC3.60 Ca^{2+} imaging lines exhibit wild type CO_2 avoidance; (B – D) AFD is a non-habituating CO_2 sensor and AFD and ASE are intrinsically CO_2 sensitive; (E – F) The AFD neurons respond to CO_2 below the cultivation temperature

A. Animals expressing the ratiometric Ca^{2+} sensor cameleon YC3.60 in AFD, BAG, ASE or AQR, PQR, URX avoid CO_2 in a 5 – 0% CO_2 gradient like wild type. Significance comparisons against N2 wild type. n = 9 assays each.

B. Mean % $\Delta R/R_0$ values for AFD OFF-responses to 3% CO_2 stimuli repeated 4 times. n = 9 traces.

C. Mean % $\Delta R/R_0$ values for AFD responses in *unc-13* mutants. Asterisks indicate significance compared to wild type. N2, n = 26 traces; *unc-13*, n = 11 traces.

D. Mean % $\Delta R/R_0$ values for ASEL responses in *unc-13* mutants. Asterisks indicate significance compared to wild type. N2, n = 18 traces; *unc-13*, n = 20 traces.

E. Mean AFD responses at 22 °C and at 15 °C in animals grown at 22 °C. AFD T = 22 °C, n = 14 traces; AFD T = 15 °C, n = 15 traces.

F. Mean % $\Delta R/R_0$ values for AFD responses at 22 °C and at 15 °C using data in (E). Asterisks indicate significance compared to T = 22 °C.

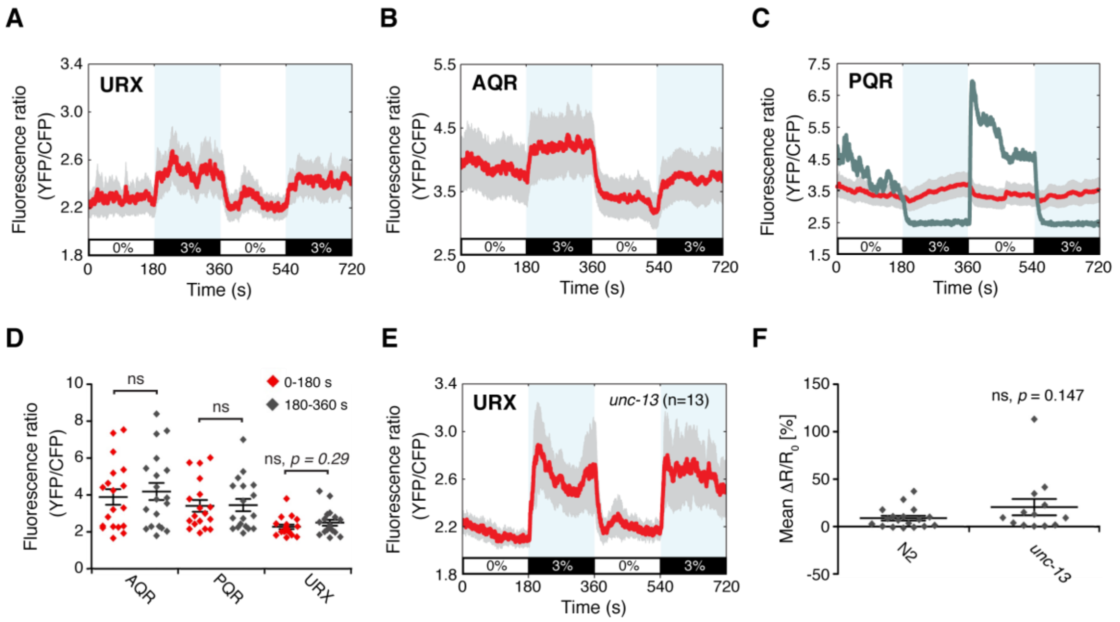

Supplementary Figure 2. The AQR, PQR and URX O_2-sensitive neurons are weakly CO_2 sensitive

A – C. Average CO_2 responses of URX (A), AQR (B) and PQR (C). The evergreen trace of panel C represents a single recording from PQR over a 21 – 11 – 21 – 11% O_2 stimulus following the timeline of the CO_2 stimulus bar. URX, n = 18 traces; AQR, n = 19 traces; PQR, n = 18 traces.

D. Time-averaged fluorescence ratios (YFP/CFP) of individual AQR, PQR and URX traces 180s before and after a 0 – 3% CO_2 increase. Horizontal bars indicate mean YFP/CFP ratio and error bars indicate SEM. ns, not significantly different before compared with after gas shift.

E. Mean URX CO_2 response in *unc-13* mutants. n = 13 traces.

F. Mean % $\Delta R/R_0$ values for URX in *unc-13* mutants. ns, indicates not significantly different from wild type. N2, n = 18 traces; *unc-13*, n = 13 traces.

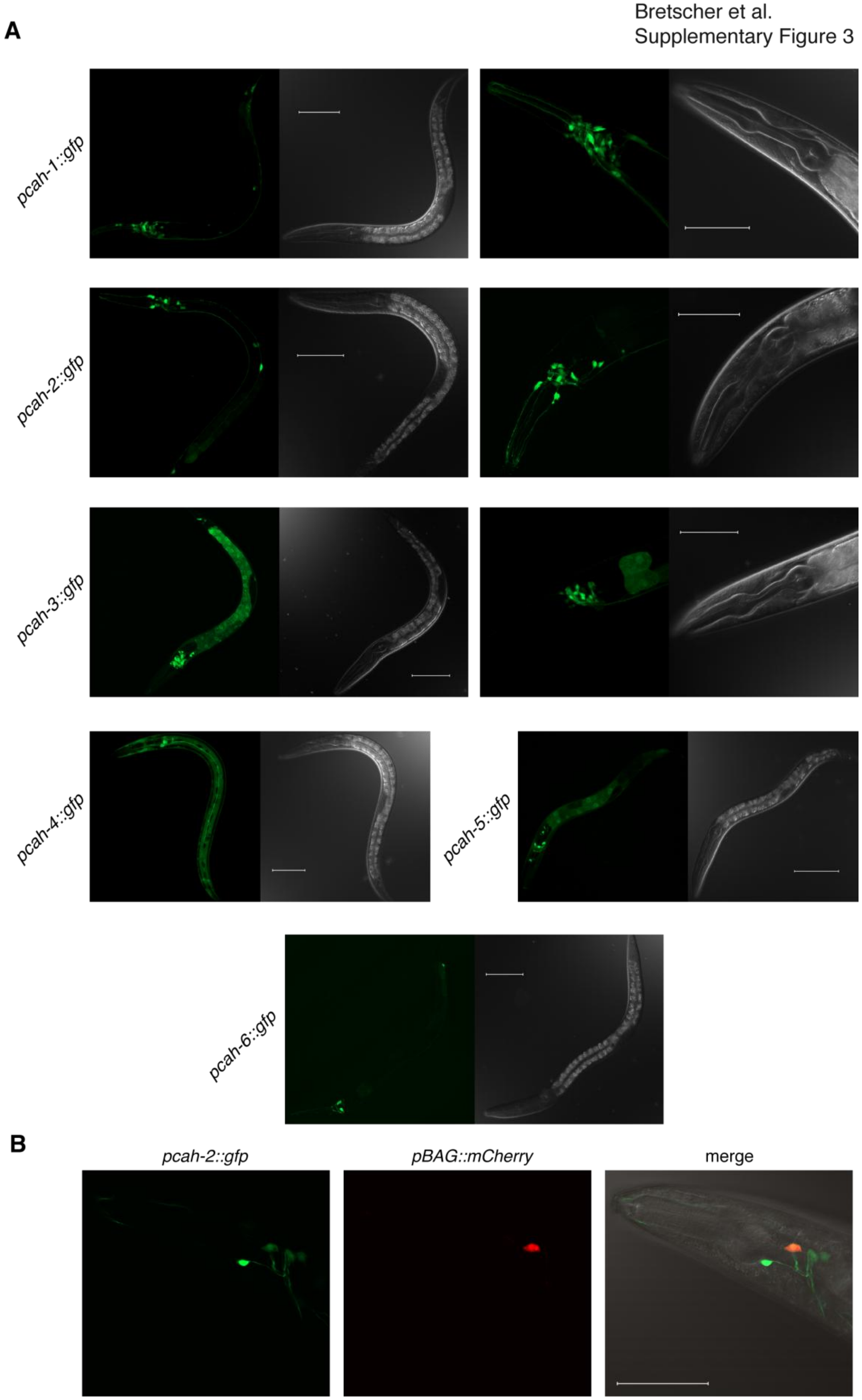
A
pcah-1::gfp
pcah-2::gfp
pcah-3::gfp
pcah-4::gfp
pcah-5::gfp
pcah-6::gfp
B
pcah-2::gfp
pBAG::mCherry
merge

Supplementary Figure 3. Expression patterns of the six *C. elegans* α-carbonic anhydrase family members

A. Upstream promoter regions from *cah-1*, *2*, *3*, *4*, *5* and *6* fused to *gfp* reveal differential expression of *cah* genes in head and tail neurons, the intestine, the hypodermis and the excretory cell. Shown are confocal *gfp* z projections and transmitted white light images of L2 animals and of young adult heads for *cah-1*, *2* and *3*. *cah-1::gfp* labels ciliated head sensory neurons, head ganglia, the ventral nerve cord and tail neurons. *cah-2::gfp* labels a set of 4 quadrant neurons in the head, BAGL/R (see (B), below), other head neurons, the mid-body canal neurons CANL and CANR and tail neurons. *cah-3::gfp* intensely labels head neurons (note these are likely interneurons as *gfp*-filled dendrites appear absent), the ventral nerve cord and the intestine. *cah-4*::gfp strongly labels the hypodermis (excluding the seam cells) and the excretory cell. *cah-5::gfp* labels head neurons and the intestine in L2 animals but neuronal expression in the head was absent by the young adult stage (not shown). *cah-6::gfp* strongly labels two bilateral pairs of head neurons and a pair of tail neurons in L2 animals and in adults (not shown). Scale bar, 50 μm.

B. The α-carbonic anhydrase *cah-2* is expressed in the BAG neurons. Shown is a confocal plane through the head of an adult animal expressing *gfp* under control of the *cah-2* promoter and *mCherry* in BAG under control of the *gcy-33* promoter. A merge of the red and green light channels shows that *gfp* and *mCherry* overlap in BAG (7/9 animals). In similar experiments, 0/12 *pcah-1::gfp* animals, 0/10 *pcah-3::gfp* animals, 0/5 *pcah-4::gfp* animals, 0/5 *pcah-5::gfp* animals and 0/9 *pcah-6::gfp* animals showed *gfp* overlap with *mCherry* in BAG. Scale bar, 50 μm.

Bretscher et al.
Supplementary Figure 4

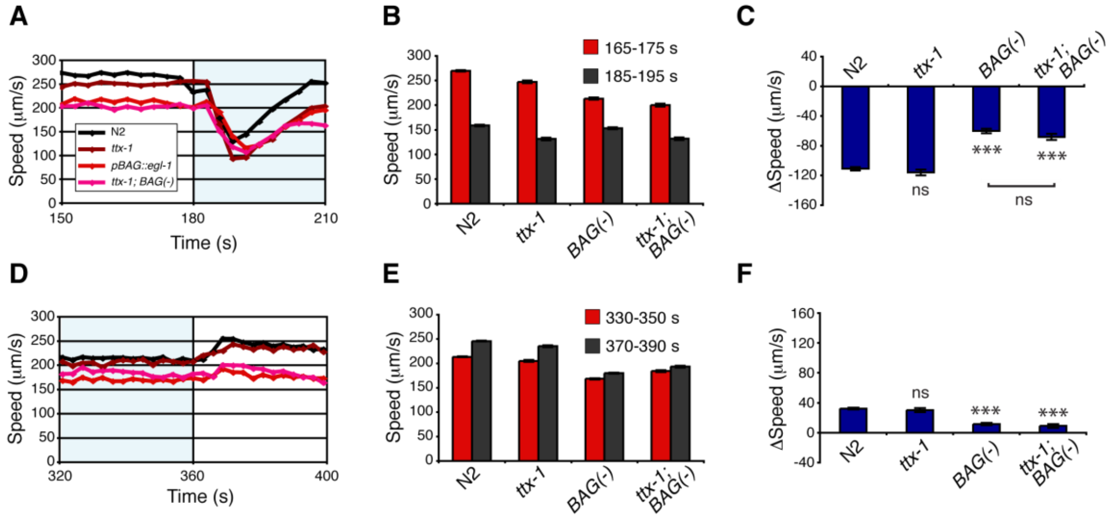

Supplementary Figure 4. Ablation of BAG reduces changes in locomotory speed across % CO_2 increases and decreases

A – F. Statistical analysis of changes in speed across 0 – 5% CO_2 increases and 5 – 0% CO_2 decreases of wild type and neuron-ablated animals off food. Average speed traces, left, time-averaged speeds before and after gas switches, middle, and average changes in speed across gas switches, right. (A, D) Average speed during 0 – 5% and 5 – 0% gas switches. Error bars omitted for clarity. (B, E) Time-averaged speeds before (red bars) and after (dark grey bars) an increase (B) or decrease (E) in % CO_2. Intervals for comparison coincide with peaks in wild type behaviour. Error bars indicate SEM. (C, F) Average change in speed across an increase (C) or a decrease (F) in % CO_2. Error bars indicate SEMs calculated from SEM values in (B, E) using error propagation formulae. Significance markers indicate comparisons with wild type, unless otherwise indicated.

Bretscher et al.
Supplementary Figure 5

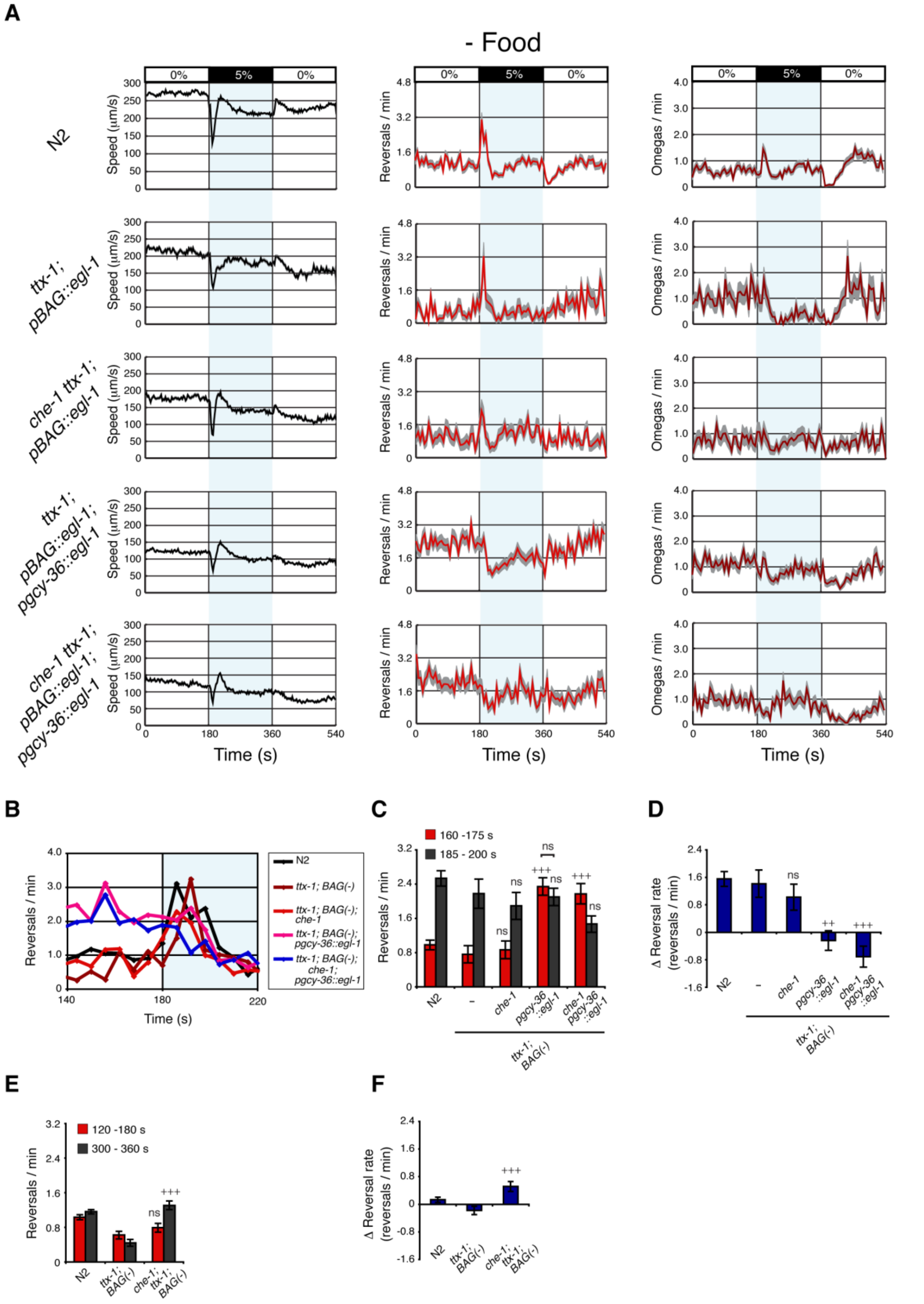

Supplementary Figure 5. The ASE and AQR, PQR and URX neurons act to inhibit reversal rate in high and low CO_2, respectively

A. Average speed, reversal and omega rates of wild type (N2), AFD-ablated BAG-ablated (*ttx-1; pgcy-33::egl-1*), AFD-, BAG-, ASE-ablated (*che-1; ttx-1; pgcy-33::egl-1*), AFD-, BAG-, AQR-, PQR- and URX-ablated (*ttx-1; pgcy-33::egl-1; pgcy-36::egl-1*) and AFD-, BAG-, ASE-, AQR-, PQR- and URX-ablated (*che-1*; *ttx-1; pgcy-33::egl-1; pgcy-36::egl-1*) animals off food across a 0 – 5 – 0% CO_2 stimulus. *che-1* encodes a transcription factor expressed only in ASE required for late ASE specification (Uchida et al., 2003; Etchberger et al., 2007). *che-1(p680)* null mutants used here phenocopy ASE laser ablated animals with respect to NaCl chemotaxis (Uchida et al., 2003; Bargmann and Horvitz, 1991; Hukema et al., 2006; Ortiz et al., 2009; Iino and Yoshida, 2009). N2, n = 59 movies; *ttx-1; pgcy-33::egl-1*, n = 16 movies; *che-1; ttx-1; pgcy-33::egl-1*, n = 19 movies; *ttx-1; pgcy-33::egl-1; pgcy-36::egl-1*, n = 19 movies; *che-1*; *ttx-1; pgcy-33::egl-1; pgcy-36::egl-1*, n = 18 movies.

B – D. Statistical analysis of changes in reversal rate across a 0 – 5% CO_2 increase for animals in (A). Average reversal rates, left, time-averaged reversal rates before and after gas switches, middle, and average change in reversal rate across gas switches, right. Rates are in reversal initiations per animal per minute. (B) Average reversal rate during a 0 – 5% gas switch. Error bars omitted for clarity. (C) Time-averaged reversal rates before (red bars) and after (dark grey bars) a 0 – 5% CO_2 increase. Error bars indicate SEM. (D) Average change in reversal rate across an increase in % CO_2. Error bars indicate compound SEMs calculated from SEM values in (C) using error propagation formulae. Significance markers (C and D) indicate comparisons against *ttx-1; BAG(-)* animals.

E – F. Statistical analysis of reversal rates across a 0 – 5% CO_2 increase of select animals in (A). (E) Time-averaged reversal rates before (red bars, 120-180s) and after (dark grey bars, 300-360s) an increase in % CO_2. Error bars indicate SEM. (F) Average change in reversal rate between the two time-points in (E). Error bars indicate compound SEMs. Significance markers indicate comparisons against *ttx-1; BAG(-)* animals.

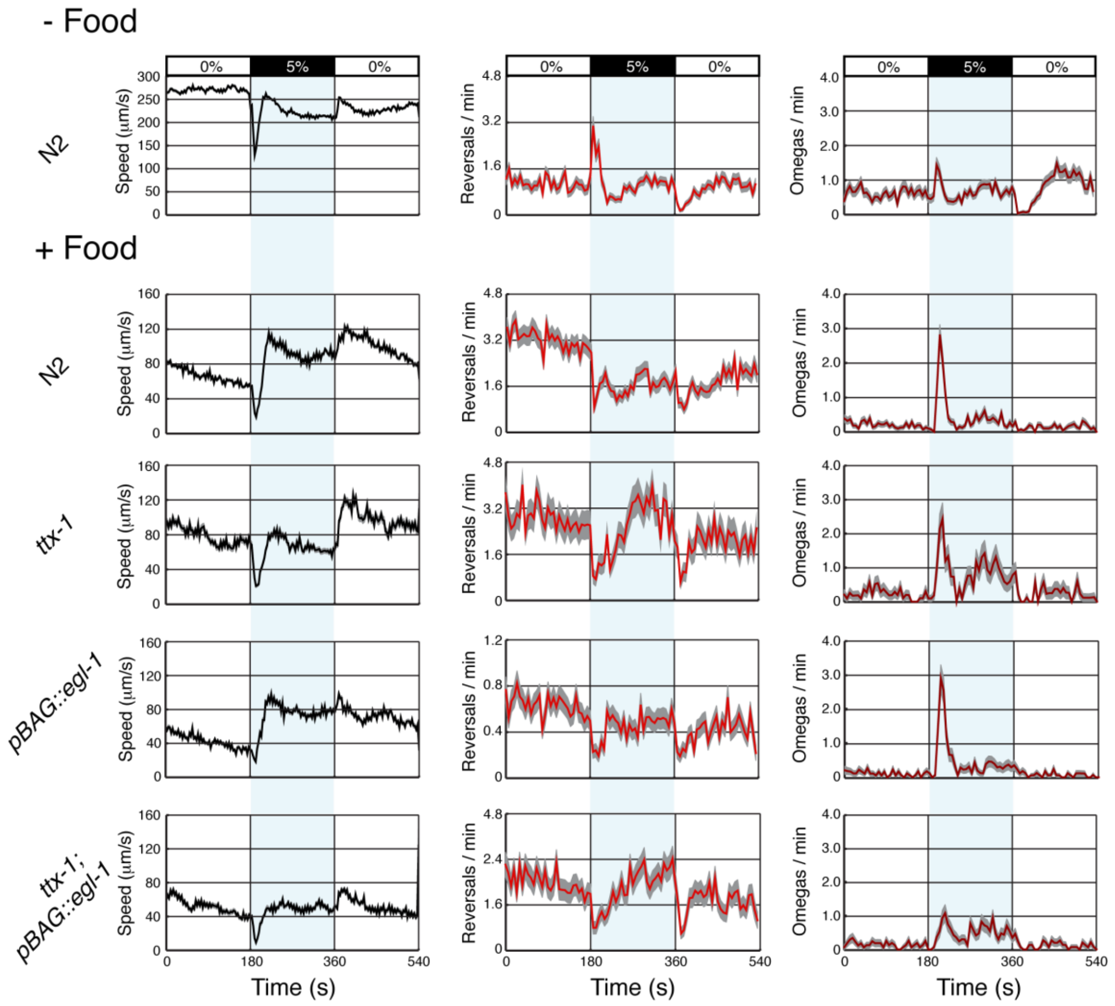

Supplementary Figure 6. AFD and BAG neurons also regulate response to % CO_2 when animals are on food

Average speed, reversal initiation and omega initiation rates of wild type (N2) on and off food and AFD-ablated (*ttx-1*), BAG-ablated (*pgcy-33::egl-1*) and AFD-ablated BAG-ablated (*ttx-1; pgcy-33::egl-1*) animals on food across a 0 – 5 – 0% CO_2 stimulus. N2 off food, n = 59 movies; N2 on food, n = 23 movies; *ttx-1(p767)*, n = 9 movies; *pgcy-33::egl-1*, n = 9 movies; *ttx-1; pgcy-33::egl-1*, n = 9 movies.

Supplementary Table 1

Plate Number	BAG (-)	BAG (+)	SUM	% BAG (-)
**1**	24	1	25	**0.96**
**2**	3	0	3	**1.00**
**3**	9	3	12	**0.75**
**4**	7	0	7	**1.00**
**5**	43	5	48	**0.90**
**6**	4	0	4	**1.00**
**7**	20	2	22	**0.91**
**8**	19	1	20	**0.95**
**9**	12	1	13	**0.92**
**10**	33	3	36	**0.92**
**11**	19	4	23	**0.83**
**12**	9	1	10	**0.90**
**13**	16	0	16	**1.00**
**14**	55	7	62	**0.89**
**15**	8	0	8	**1.00**
**16**	35	4	39	**0.90**
**17**	24	0	24	**1.00**
**18**	17	2	19	**0.89**
**19**	28	6	34	**0.82**
**20**	3	0	3	**1.00**
**21**	34	2	36	**0.94**
**22**	16	2	18	**0.89**
**23**	5	0	5	**1.00**
**24**	9	1	10	**0.90**
**TOTALS**	**452**	**45**	**497**	——
**The Mean**	——	——	——	**0.93**
**The SEM**	——	——	——	**0.013**

Supplementary Table 1. Quantification of BAG ablation by the *pgcy-33:egl-1* transgene

Animals from the strain AX2175, *lin-15(n765ts) X; dbEx [pgcy-33::egl-1, ccdsRed], dbEx [pflp-17::yc360, lin-15(+)]* that were both *dsRed(+)* and Non-Muv were scored for green fluorescent BAG neurons on 3 separate days. Such animals carry both *pflp-17::yc360*, that labels BAG green fluorescent, and *pgcy-33::egl-1*. Non-Muv animals that are *dsRed(+)* but not *YC3.60(+)* were scored as having lost BAGL/R. Note, ~ 100% of Non-Muv animals from the strain AX2073, *lin-15(n765ts) X; dbEx [pflp-17::yc360, lin-15(+)]* express YC3.60 in BAG (not shown).

Supplementary Experimental Procedures

Strains

In this study *C. elegans* Bristol strain N2 was used as the reference wild type. Other strains used, listed in order of appearance are; PR691, *tax-2(p691) I*; CX2948, *tax-4(p678) III*; AX1960, *tax-2(p691) I; tax-4(p678) III*; OH150, *tax-2(ot25) I*; PR694, *tax-2(p694) I*; AX2120, *tax-2(p694) I; dbEx [Bgl II – Hind III 8.4 Kb genomic fragment, pelt-2:gfp]*; AX2164, *tax-2(p694) I; lin-15(n765ts) X; dbEx [pgcy-8::, pgcy-32::, pflp-17::, pflp-6::tax-2 cDNA::SL2gfp, lin-15(+)]*; AX2178, *tax-2(p694) I; lin-15(n765ts) X; dbEx [pgcy-8::tax-2 cDNA::SL2gfp, lin-15(+)]*; AX2157, *tax-2(p694) I; lin-15(n765ts) X; dbEx [pflp-17::tax-2 cDNA::SL2gfp, lin-15(+)]*; AX2161, *tax-2(p694) I; lin-15(n765ts) X; dbEx [pflp-6::tax-2 cDNA::SL2gfp, lin-15(+)]*; AX2159, *tax-2(p694) I; lin-15(n765ts) X; dbEx [pgcy-32::tax-2 cDNA::SL2gfp, lin-15(+)]*; AX2047, *dbEx [pgcy-8::YC3.60, podr-1::mCherry]*; AX2112, *unc-13(e51) I*; *dbEx [pgcy-8::YC3.60, podr-1::mCherry]*; AX2073, *lin-15(n765ts) X; dbEx [pflp-17::YC3.60, lin-15(+)]*; AX2099, *unc-13(e51) I; dbEx [pflp-17::YC3.60, lin-15(+)]*; AX2102, *unc-31(e928) IV; dbEx [pflp-17::YC3.60, lin-15(+)]*; XL76, *ntIs13[pflp-6::YC2.12, lin-15(+)]*; AQ1044, *lin-15(n765ts) X; ljEx95 [psra-6::YC2.12, lin-15(+)]*; IK597, *gcy-8(nj37) gcy-18(oy44) gcy-23(nj38) IV*; AX2054, *gcy-31(ok296) X; gcy-33(ok232) V*; AX2135, *gcy-31(ok296) X; gcy-33(ok232) V; dbEx [pflp-17::YC3.60, lin-15(+)]*; AX2121, *tax-2(p694) I; dbEx [pflp-17::YC3.60, lin-15(+)]*; AX2166, *tax-4(p678) III; dbEx [pflp-17::YC3.60, lin-15(+)]*; AX2125, *ttx-1(p767) V*; PY2137, *ttx-1(p767) V; Ex [14 Kb ttx-1(+), punc-122::gfp]*; AX2051, *dbEx [pgcy-33::egl-1, punc-122::dsRed]*; AX2172, *ttx-1(p767) V; dbEx [pgcy-33::egl-1, punc-122::dsRed]*; AX2175, *lin-15(n765ts) X; dbEx [pflp-17::YC3.60, lin-15(+)]; dbEx [pgcy-33::egl-1, punc-122::dsRed]*; AX1904, *dbEx [pgcy-32::YC3.60, lin-15(+)]*; AX1965, *unc-13(e51) I; dbEx [pgcy-32::YC3.60, lin-15(+)]*; AX2467, *lin-15(n765ts) X; dbEx [pcah-1::gfp, lin-15(+)]*; AX2447, *lin-15(n765ts) X; dbEx [pcah-2::gfp, lin-15(+)]*; AX2450, *lin-15(n765ts) X; dbEx [pcah-3::gfp, lin-15(+)]*; AX2470, *lin-15(n765ts) X; dbEx [pcah-4::gfp, lin-15(+)]*; AX2473, *lin-15(n765ts) X; dbEx [pcah-5::gfp, lin-15(+)]*; AX2453, *lin-15(n765ts) X; dbEx [pcah-6::gfp, lin-15(+)]*; AX2458, *lin-15(n765ts) X; dbEx [pgcy-33::mCherry, pcah-2::gfp, lin-15(+)]*; AX2415, *che-1(p680) I; ttx-1(p767) V; dbEx [pgcy-33::egl-1, punc-122::dsRed]*; AX2442, *ttx-1(p767) V; qaIs2241 [pgcy-36::egl-1, pgcy-*

35::gfp, lin-15(+)]; dbEx [pgcy-33::egl-1, punc-122::dsRed]; AX2444, *che-1(p680) I; ttx-1(p767) V; qaIs2241 [pgcy-36::egl-1, pgcy-35::gfp, lin-15(+)]; dbEx [pgcy-33::egl-1, punc-122::dsRed].*

Transgenic strain construction

Expression constructs were made using the Invitrogen Multisite Gateway 3-Fragment Vector Construction Kit. This enables any 5’ gene element (site 1) to be recombined with any open reading frame (site 2) and any 3’ gene element (site 3) in a one-step plasmid LR (Lambda Recombinase) reaction. For *tax-2* rescues, promoters (site 1), a full-length *tax-2* cDNA (site 2) and SL2-*gfp* (site 3) were inserted into a modified Gateway destination plasmid containing the *unc-54* 3’ UTR. When all elements are recombined, a polycistronic gene is formed that co-expresses the *tax-2* and *gfp* genes, enabling confirmation of expression (Coates and de Bono, 2002). To drive expression, we used an AFD-specific 2.2 Kb *gcy-8* promoter fragment (Yu et al., 1997), a BAG-specific 3.3 Kb *flp-17* promoter fragment (Kim and Li, 2004), an ASE-specific 1.9 Kb *flp-6* promoter fragment (Suzuki et al., 2008) and an AQR, PQR, URX-specific 0.6 Kb *gcy-32* promoter fragment (Yu et al., 1997). Primer details below. Non-Muv *tax-2(p694) lin-15(n765ts)* hermaphrodites grown at 15 °C were transformed by microinjection (Mello et al., 1991) of mixtures containing expression construct at 20 ng/μl, pJMZ-*lin-15* construct at 50 ng/μl and pBluescript (‘stuffer’ DNA) at 30 ng/μl in 5 mM Tris-Cl pH 8.0. For injection of all 4 *promoter::tax-2* constructs together, a solution containing each construct at 20 ng/μl plus 50 ng/μl pJMZ- *lin-15* but no pBluescript was used. For cameleon YC3.60 expression in BAG, *lin-15(n765ts)* animals were injected. Expression studies of YC3.60 in BAG revealed that introduction of the splice leader 1 (SL1) sequence TTTTCAG and a kozak consensus 5’ of the YC3.60 ORF improved expression. The *pflp-6::YC2.12* ASE and *pgcy-8::YC3.60* AFD imaging lines were received from Bill Schafer and Piali Sengupta, respectively.

For expression studies of the six *C. elegans* α-carbonic anhydrase family members *cah-1* to cah-6, promoter fragments were cloned into Gateway expression vectors together with *gfp* and the *unc-54* 3’ UTR. Promoter fragments from the *cah-2*, *cah-3* and *cah-6* genes were 2.0 Kb in length and ordered from the *C. elegans*

Promoterome Project (Source Bioscience and Dupuy et al., 2004). Promoter fragments for *cah-1*, *cah-4* and *cah-5* were 5.5 Kb, 4.1 Kb and 1.5 Kb, respectively.

Sequences of forward and reverse primers for promoter cloning, including the *attB4* and *attB1* Gateway sequences (upper case):

attB4pflp17F GGGGACAACTTTGTATAGAAAAGTTGccttgaagcttttcctctg
attB1pflp17R GGGGACTGCTTTTTTGTACAAACTTGctggaaaaataaagttttgcg
attB4pgcy8F GGGGACAACTTTGTATAGAAAAGTTGagcaaagggcgtcgattatctc
attB1pgcy8R GGGGACTGCTTTTTTGTACAAACTTGtttgatgtggaaaaggtagaatc
attB4pflp6F GGGGACAACTTTGTATAGAAAAGTTGccatcttcacgtcacttctgc
attB1pflp6R GGGGACTGCTTTTTTGTACAAACTTGtcagctatacgtctgcgaagg
attB4pcah1F GGGGACAACTTTGTATAGAAAAGTTGgaagtataagtgtcgcagatttg
attB1pcah1R GGGGACTGCTTTTTTGTACAAACTTGaggagatctgaattattgaattg
attB4pcah4F GGGGACAACTTTGTATAGAAAAGTTGgtggttgacctagttgcctgaattg
attB1pcah4R GGGGACTGCTTTTTTGTACAAACTTGtttttctttcgtcggtcttttggtc
attB4pcah5F GGGGACAACTTTGTATAGAAAAGTTGcaaatccagtgcaacaaacagag
attB1pcah5R GGGGACTGCTTTTTTGTACAAACTTGaggagcaagatgtcttggtgttc

Fabrication of Microfluidic Devices

Microfluidic devices were fabricated from polydimethylsiloxane (PDMS) using standard soft lithography technology (Xia and Whitesides, 1998; Qin et al., 2010). Devices were designed in AutoCAD (Autodesk) and printed on a film photomask (Photo Data Ltd, UK) at 128000 dots per inch (dpi). A master mould was created by spin coating a 200 μm thick layer of SU-8 2150 photoresist (MicroChem) at 2000 rpm for 30 s onto a silicon wafer, which was then patterned by photolithography. A 3 mm thick layer of PDMS prepolymer mixture (Sylgard 184, Dow Corning) was poured over the mould and cured for 1 day at RT to create the device. Inlets and outlet holes were punctured into the PDMS using 20 gauge stub adapters (Intramedic).

Details of temperature controlled Ca^{2+} imaging

For imaging AFD at different temperatures (Supplementary Figure 1E, F), a temperature-controlled brass stage was built with inlayed peltier blocks, water-cooling system and thermocouple sensor. A switch-board interface plugged into the mains

powered the peltier blocks and thermocouple sensor and received commands from a computer via an RS232 link. Running a simple custom-written programme ‘Temperature Set’ for PC enabled temperature-control of the brass stage via feedback from the thermocouple sensor.

Confocal Imaging

Confocal imaging was done on a Zeiss LSM 510 laser scanning confocal microscope using a 40x EC Plan-Neofluar/1.30 NA oil objective and a pinhole of 0.92 Airy Units. Worms were mounted on 2% agarose pads made up in 10 mM sodium azide and M9 Buffer. For *gfp* imaging, a 488 nm laserline from an Argon laser was used with a BP 505-570 band pass emission filter. For *mCherry* imaging, 543 nm excitation light from a HeNe laser was used with an LP 560 long-wave pass emission filter. Pixel brightness adjustment of the *gfp* image by a gamma correction of 1.25, cropping and resizing was carried out in Adobe Photoshop Version 10.0.1.

Determination of the *tax-2(p694)* deletion limits

The *tax-2(p694)* mutant strain PR694 was obtained from the *Caenorhabditis* Genetics Centre. The forwards primer p694F4 GTTGATCGGTTGACAATCAGTAG and reverse primer p694R4 GCTCGAAGTAGCCCAAACATTTC amplify a 2.2 Kb fragment across the *p694* lesion. Sequencing this PCR product with 4 internal primers revealed *p694* to be a 390 bp deletion and a 36 bp insertion, compared with the previously reported ~ 1.6 Kb deletion (Coburn and Bargmann, 1996). *p694* removes 264 bp of sequences upstream of *tax-2*, exon 1 (81 bp), 45 bp of the first intron and introduces a 36 bp A/T rich insert. This definition is confirmed by primers p694F5 ATGATGACTGCTTGGCAACGGAC and p694R4 that flank the deletion and by p694F7 TCATTTCTTTGCGTCTCCTTG that lies within the deletion. A p694F5/R4 PCR yields a 697 bp product from wild type and a 342 bp product from *p694* template. A p694F7/R4 PCR yields a 480 bp product from wild type and no product from *p694* template.

Supplementary references

Dupuy, D., Li, Q. R., Deplancke, B., Boxem, M., Hao, T., Lamesch, P., Sequerra, R., Bosak, S., Doucette-Stamm, L., Hope, I. A., Hill, D. E., Walhout, A. J., Vidal, M. (2004). A first version of the *Caenorhabditis elegans* Promoterome. Genome Res *14*, 2169-2175.

Etchberger, J. F., Lorch, A., Sleumer, M. C., Zapf, R., Jones, S. J., Marra, M. A., Holt, R. A., Moerman, D. G., and Hobert, O. (2007). The molecular signature and cis-regulatory architecture of a *C. elegans* gustatory neuron. Genes Dev *21*, 1653-1674.

Hukema, R. K., Rademakers, S., Dekkers, M. P., Burghoorn, J., and Jansen, G. (2006). Antagonistic sensory cues generate gustatory plasticity in *Caenorhabditis elegans*. EMBO J *25*, 312-322.

Iino, Y., and Yoshida, K. (2009). Parallel use of two behavioral mechanisms for chemotaxis in *Caenorhabditis elegans*. J Neurosci *29*, 5370-5380.

Kim, K., and Li, C. (2004). Expression and regulation of an FMRFamide-related neuropeptide gene family in *Caenorhabditis elegans*. J Comp Neurol *475*, 540-550.

Mello, C. C., Kramer, J. M., Stinchcomb, D., and Ambros, V. (1991). Efficient gene transfer in *C. elegans*: extrachromosomal maintenance and integration of transforming sequences. EMBO J *10*, 3959-3970.

Qin, D., Xia, Y., and Whitesides, G. M. (2010). Soft lithography for micro- and nanoscale patterning. Nat Protoc *5*, 491-502.

Uchida, O., Nakano, H., Koga, M., and Ohshima, Y. (2003). The *C. elegans che-1* gene encodes a zinc finger transcription factor required for specification of the ASE chemosensory neurons. Development *130*, 1215-1224.

Xia, Y., and Whitesides, G. (1998). Soft Lithography. Annual Review of Materials Science *28*, 153-184.
