# Supplementary figures and images for: Temperature, Oxygen, and Salt-Sensing Neurons in C. elegans Are Carbon Dioxide Sensors that Control Avoidance Behavior
Source: Neuron. 2011 Mar 24;69(6-4):1099–113. doi: 10.1016/j.neuron.2011.02.023 (PMC3115024; doi:10.1016/j.neuron.2011.02.023)

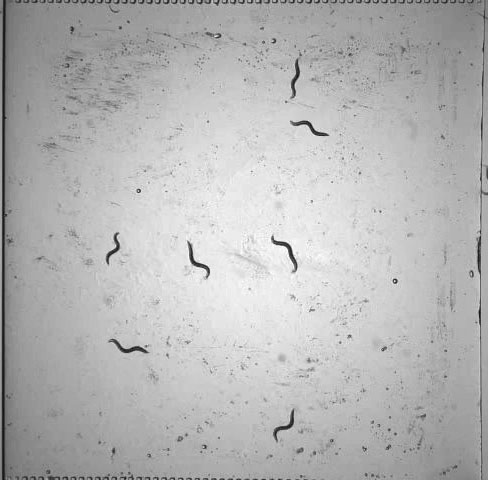

Supplement: Movie S1. C. elegans Responses to a 0%–5%–0% CO2 Stimulus following a 1–3–1 min Timeline off Food — Animals placed within the square chamber microfluidic device are exposed to 5% CO2 stimuli off food. Movie speeded up 5×. Timing of CO2 stimulus is indicated. [file mmc2.jpg]

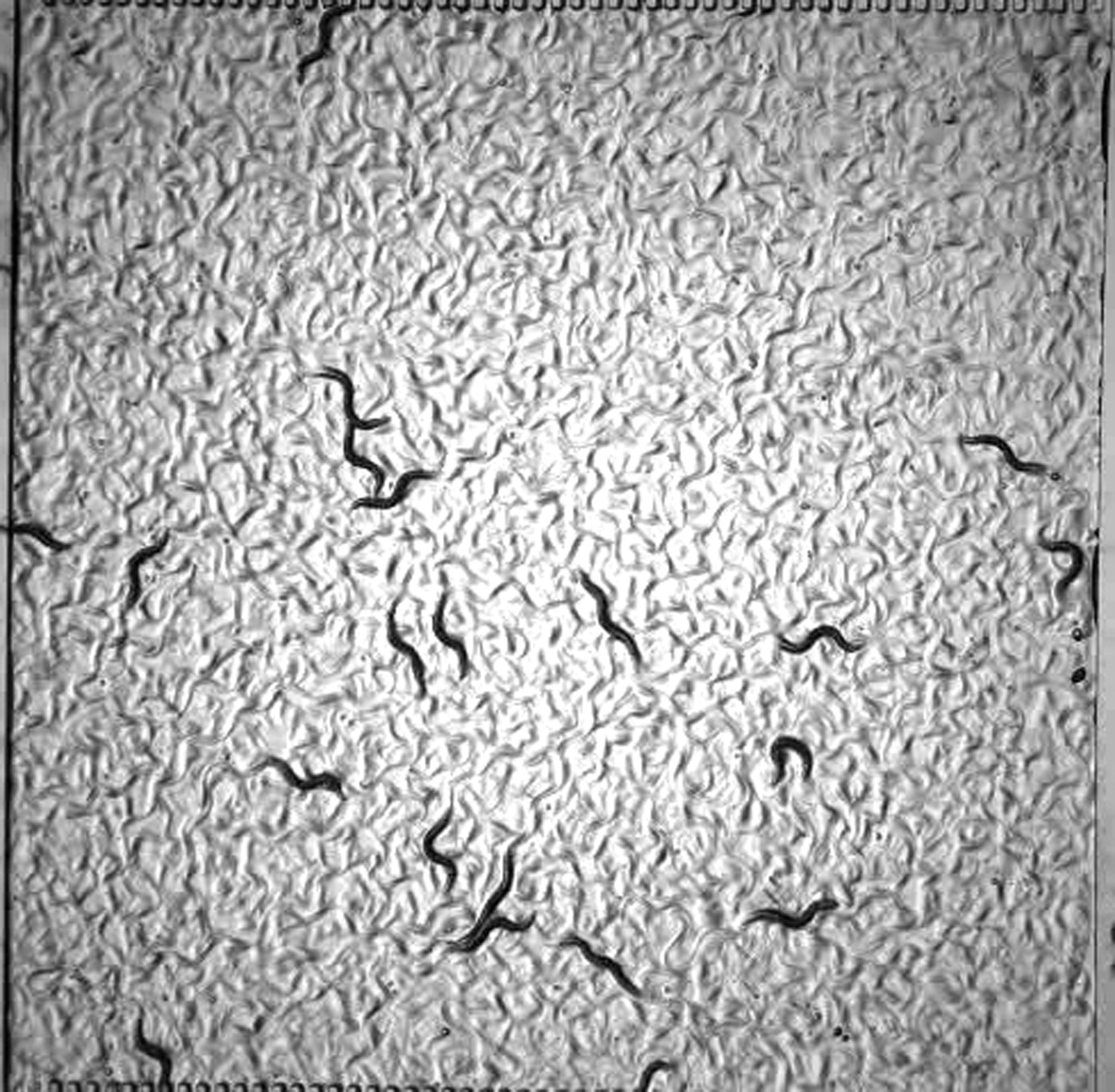

Supplement: Movie S2. Responses of Feeding C. elegans to a 0%–5%–0% CO2 Stimulus following a 1–3–1 min Timeline — As in Movie S1, but animals are exposed to 5% CO2 while on a lawn of E. coli food. Movie speeded up 5×. Timing of CO2 stimulus is indicated. [file mmc3.jpg]
